# Efficacy and safety of methylene blue in the treatment of malaria: a systematic review

**DOI:** 10.1186/s12916-018-1045-3

**Published:** 2018-04-25

**Authors:** G. Lu, M. Nagbanshi, N. Goldau, M. Mendes Jorge, P. Meissner, A. Jahn, F. P. Mockenhaupt, O. Müller

**Affiliations:** 1 Medical College of Yangzhou University, Yangzhou University, Yangzhou, 225001 China; 2 Institute of Public Health, Medical School, Ruprecht-Karls-University Heidelberg, Heidelberg, Germany; 3 Department of Paediatric and Adolescent Medicine, Ulm University, Ulm, Germany; 4 Institute of Tropical Medicine and International Health, Charité-Universitätsmedizin Berlin, Berlin, Germany

**Keywords:** Malaria, Methylene blue, Efficacy and safety, Drug resistance, Elimination

## Abstract

**Background:**

Methylene blue (MB) was the first synthetic antimalarial to be discovered and was used during the late 19th and early 20th centuries against all types of malaria. MB has been shown to be effective in inhibiting *Plasmodium falciparum* in culture, in the mouse model and in rhesus monkeys. MB was also shown to have a potent ex vivo activity against drug-resistant isolates of *P. falciparum* and *P. vivax*. In preclinical studies, MB acted synergistically with artemisinin derivates and demonstrated a strong effect on gametocyte reduction in *P. falciparum*. MB has, thus, been considered a potentially useful partner drug for artemisinin-based combination therapy (ACT), particularly when elimination is the final goal. The aim of this study was to review the scientific literature published until early 2017 to summarise existing knowledge on the efficacy and safety of MB in the treatment of malaria.

**Methods:**

This systematic review followed PRISMA guidelines. Studies reporting on the efficacy and safety of MB were systematically searched for in relevant electronic databases according to a pre-designed search strategy. The search (without language restrictions) was limited to studies of humans published until February 2017.

**Results:**

Out of 474 studies retrieved, a total of 22 articles reporting on 21 studies were eligible for analysis. The 21 included studies that reported data on 1504 malaria patients (2/3 were children). Older studies were case series and reports on MB monotherapy while recent studies were mainly controlled trials of combination regimens. MB was consistently shown to be highly effective in all endemic areas and demonstrated a strong effect on *P. falciparum* gametocyte reduction and synergy with ACT. MB treatment was associated with mild urogenital and gastrointestinal symptoms as well as blue coloration of urine. In G6PD-deficient African individuals, MB caused a slight but clinically non-significant haemoglobin reduction.

**Conclusions:**

More studies are needed to define the effects of MB in *P. falciparum* malaria in areas outside Africa and against *P. vivax* malaria. Adding MB to ACT could be a valuable approach for the prevention of resistance development and for transmission reduction in control and elimination programs.

**Systematic review registration:**

This study is registered at PROSPERO (registration number CRD42017062349).

**Electronic supplementary material:**

The online version of this article (10.1186/s12916-018-1045-3) contains supplementary material, which is available to authorized users.

## Background

Malaria remains the most important parasitic disease in humans [1]. Combination treatment of malaria has become the accepted paradigm in malaria control, with the particular aim of delaying and possibly reversing the development of drug resistance [2]. Artemisinin-based combination therapy (ACT) has become the standard treatment for falciparum malaria in virtually all endemic regions in the 21st century [3, 4]. However, artemisinin resistance is emerging in South-East Asia, and there is a clear need to develop strategies to protect the ACTs [5]. One potentially useful strategy could be to add a third drug with an independent anti-parasitic activity [6]. Moreover, the World Health Organization recommends the addition of a gametocytocidal drug such as primaquine (PQ) to current ACT regimens for malaria elimination programs [7].

Methylene blue (MB) is a water-soluble dye, which has been used for a long time in industry and medicine [8, 9]. The drug is rapidly and widely distributed throughout the body [10, 11]. The drug is well absorbed from the gastrointestinal tract and partly monodemethylated to azure B. Maximal plasma concentrations are reached 2 hours following oral administration, and the plasma half-life is about 20 h [12]. Renal excretion of MB and azure B (in oxidised blue or reduced uncoloured forms) is the main elimination pathway [13, 14].

MB is a registered drug in most countries for various indications, such as the treatment of both acquired and hereditary methaemoglobinaemia, the prevention of ifosfamide-induced encephalopathy in human cancer management, the prevention of urinary tract infections, the intraoperative visualisation of nerves and endocrine glands as well as of pathologic fistulae, and the sterilisation of transfusion blood [9, 15–23]. MB has also been considered to be effective in priapism, against septic shock (blocking the NO-dependant guanylate-cyclase) and in vasoplegic patients after cardiac surgery, and it is under investigation as an experimental drug against Alzheimer’s disease [24–28]. MB was the first synthetic antimalarial to be used, which occurred in a German hospital some 120 years ago [29]. Its global use in malaria endemic areas is well documented for the late 19th and early 20th centuries [30, 31]. However, it stopped being used after new synthetic antimalarials were developed [9].

The interest in MB as an antimalarial drug was reactivated when *Plasmodium falciparum* glutathione reductase was identified as a new drug target [8, 32–34], although this concept has been questioned by other experimental evidence [35]. Like its major catabolite, azure B, MB is a subversive redox-cycling substrate, and like 4-aminoquinolines, it also interacts with the polymerisation of haem to hemozoin [34, 36–38]. Indeed, further but not yet fully understood mechanisms appear to be involved, which renders the development of resistance to MB with its multifactorial activity rather unlikely [9, 10]. The development of resistance to MB was proven to be difficult in vivo [39]. MB has been shown to be effective in inhibiting *P. falciparum* in culture, in the mouse model and in rhesus monkeys [40, 41]. MB was also shown to have a potent ex vivo activity against drug-resistant isolates of *P. falciparum* and *P. vivax* [42, 43]. In preclinical studies, MB acted synergistically with artemisinin derivates, but not with chloroquine (CQ), and was shown to have a strong effect on gametocyte reduction in *P. falciparum* [44–48]. MB has, thus, been considered a potentially useful partner drug for ACT, particularly when elimination is the final goal [49]. The aim of this study was to review the scientific literature published until early 2017 to summarise existing knowledge on the efficacy and safety of MB in the treatment of malaria.

## Methods

This systematic review follows the PRISMA (Preferred Reporting Items for Systematic Reviews and Meta-Analyses) guidelines [50].

### Study inclusion and exclusion criteria

All available and accessible data published until 28 February 2017 on the efficacy and/or safety of MB in the treatment of malaria were considered. Studies reporting data on MB given to humans of any age as monotherapy or in combination with other antimalarials and irrespective of drug formulations and sample size were included. In vitro and animal studies with MB, reports on the use of MB in humans for indications that were not malaria and review papers were excluded.

### Search strategy and selection criteria

Standard electronic databases were searched for scientific papers on the subject, but also hand searches were done in scientific books of major libraries. The following databases were searched for studies published in any language: Medline, Embase, BIOSIS, Cochrane Central Register of Controlled Trials, Cochrane Library, Web of Science, and the China National Knowledge Infrastructure and the Chinese Biomedical databases. The search used combinations of the terms “malaria” and “methylene blue” as both medical subject headings and key or free text words and included a broad range of derivations to ensure as wide a search strategy as possible. A list of the detailed search strategy used is available online as Additional file 1. We also retrieved and manually searched articles with relevant titles but an unclear or no abstract. Bibliographies of reports were searched and additional relevant references identified and, where appropriate, included in the review.

### Data extraction and analysis

The electronic reports identified were imported into the reference manager *Endnote* and duplicates removed. Each paper was assessed in two phases: first by screening title and abstract, and then by full-text review to ensure it met the inclusion criteria. The first assessment was done by two reviewers (MN and NG). The second assessment was done by GL and OM. An additional reviewer (AJ) settled any discordance between the reviewers.

Predetermined study characteristics were defined for extraction and documentation by two of the investigators (GL and OM), first independently and then in a consensus procedure. Reports selected were stratified according to study type. The primary outcome was the reported cure rate. Secondary outcomes were fever and parasite clearance rates, the effect on malaria gametocytes and adverse events (AEs).

### Quality appraisal

Two of the investigators (GL and OM) evaluated the quality of included studies in a consensus procedure. The risk of bias of randomised controlled trials (RCTs) was evaluated by the tool described in the Cochrane Handbook for Systematic Reviews of Interventions [51]. The risk of bias in other controlled studies was evaluated by MINORS, for which 12 methodological items are reported [52]. Each domain was scored 0 (not reported), 1 (reported but inadequate) or 2 (reported and adequate). The maximum ideal score was 24.

Quality criteria for case series and case reports were developed from MINORS and the 2016 Critical Appraisal Checklist of the Joanna Briggs Institute for case reports and case series [52–54]. Seven methodological domains were evaluated (score 0 = not reported, score 1 = reported but inadequate or score 2 = reported and adequate). The maximum ideal score was 14. Where necessary, study authors have been contacted for clarification. Due to a lack of homogeneity among the studies, a meta-analysis was not possible.

## Results

### Study selection

**Fig. 1:**
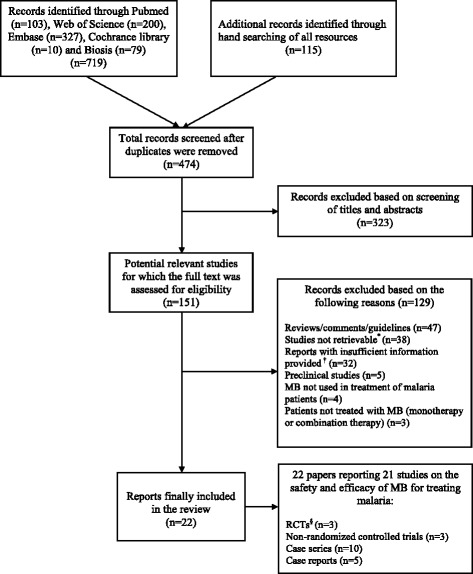
Selection process of the review. * Original full article not available due to it being very old despite exhaustive searches of all possible resources including databases and libraries and making contact with journal archivists. † Information was missing on more than three parameters of the analysis and there was no possibility of getting this from other resources. MB methylene blue, RCT randomised controlled trial

Figure 1 summarises the selection process. After removing duplicates, the literature search identified 474 records of which 151 were selected for full text assessment (Additional file 2). Of these, 129 were excluded leaving 22 publications reporting on 21 studies for data extraction and further analysis [29, 55–75].

The most common reasons for exclusion were as follows: the manuscripts were reviews or comments or guidelines (*n* = 47), studies were not retrievable despite exhaustive efforts due to very old date (*n* = 38), manuscripts provided insufficient information (*n* = 32), preclinical studies (*n* = 5), MB was not used for treatment of malaria (*n* = 4), and patients were not treated with MB (*n* = 3).

### General study characteristics

The 21 studies included are summarised in Table 1. They originated from Africa (*n* = 7), Asia (*n* = 3), the Americas (*n* = 3) and Europe (*n* = 8) and reported data on 1504 malaria patients (2/3 were children). The majority of reports were historical studies from around the year 1900 (*n* = 15), one was from the year 1949, and the remaining (*n* = 5) were published after the year 2000. The historical studies reported on malaria types diagnosed microscopically and/or clinically, which were not always well specified (of 368 cases, 30 were diagnosed as quartan malaria, 87 as tertian malaria, 183 as falciparum malaria, one as a double infection with tertian malaria and falciparum malaria, and the remaining were not specified). The more recently conducted controlled trials reported microscopically well determined outcomes on falciparum malaria (*n* = 5) and on vivax malaria (*n* = 1).

**Table 1:** Summary of included studies assessing methylene blue (MB) in the treatment of malaria

Study: First Author Place of study (Year)	Patient information	Malaria type	Malaria diagnosis	MB treatment	Follow-up	Efficacy outcome	Safety outcome	Other Information
Randomised controlled trials								
Coulibaly et al*.* Burkina Faso (2015) [55]	*n* = 193 (6–59-month-old children)	*P. falciparum*	By microscope	Arm 1: AS-AQ-MB (*n* = 92) (MB: 15 mg/kg per day for 3 days) Arm 2: AS-AQ (*n* = 101) Formulation: mini-tablets	28 days	ACPR was 80% in arm 1, and 85% in arm 2. Significant lower gametocyte prevalence on day 7 in arm 1 compared to arm 2 (both microscopically and molecular biologically) Clearance of *P. falciparum* asexual parasites in AS-AQ-MB took 1.82 days compared to 1.96 days in the AS-AQ group	MB regimen was associated with more vomiting. Haemoglobin values were significantly lower in arm 1 than in arm 2 at day 2 and day 7 (difference 0.5–1.0 mg/dl)	(1) There were no differences in parents and caregivers self-reported acceptance rate between groups (2) The MB mini-tablets were provided on a spoon with local food to improve the acceptability for children
Zoungrana et al. Burkina Faso (2008) [56, 57]	*n* = 180 (6–10-year-old children)	*P. falciparum*	By microscope	Arm 1: MB-AS (*n* = 61) Arm 2: MB-AQ (*n* = 58) (MB: 20 mg/kg per day for 3 days) Arm 3: AS-AQ (*n* = 61) Formulation: taste-masked tablets	28 days	ACPR was 62% in arm 1, 95% in arm 2 and 82% in arm 3 MB regimens were associated with a more rapid parasite clearance and significantly reduced gametocyte prevalence during follow-up	MB regimen was associated with vomiting and dysuria	Vomiting was shown to be much reduced by administering MB together with food
Meissner et al. Burkina Faso (2005) [58]	*n* = 226 (6–59-month-old children)	*P. falciparum*	By microscope	Arm 1: CQ-MB (*n* = 181) (MB: 4 mg/kg per day for 3 days) Arm 2: CQ (*n* = 45) Formulation: 0.5% MB solution	14 days	ACPR was 56% (93/166) in arm 1 compared to 46% (19/41) in arm 2	No differences in SAEs, and no cases of severe haemolysis No differences in haemoglobin over time in both the G6PD-deficient and G6PD-sufficient subgroups	Administration of the bitter-tasting MB solution was sometimes difficult, especially in younger children
Non-randomised control trials								
Bountogo et al. Burkina Faso (2010)^a^ [59]	*n* = 60 (age range: 18–55 years, median 25)	*P. falciparum*	By microscope	Arm 1: MB for 7 days (*n* = 20) Arm 2: MB for 5 days (*n* = 20) Arm 3: MB for 3 days (*n* = 20) MB: 780 mg per day Formulation: taste-masked tablets	28 days	Arm 1: 0/20 recrudescence Arm 2: 4/19 recrudescence Arm 3: 2/20 recrudescence	Dysuria (47/60). Gastrointestinal symptoms (13/60). No significant differences in adverse events between groups	MB was given at a dose of 390 mg twice daily after breakfast and supper
Meissner et al. Burkina Faso (2006)^b^ [60]	*n* = 435 (6–59-month-old children)	*P. falciparum*	By microscope	Arm 1: CQ-MB (*n* = 156) (MB: 12 mg/kg per day for 3 days) Arm 2: CQ-MB (*n* = 155) (MB: 18 mg/kg per day for 3 days) Arm 3: CQ-MB (*n* = 123) (MB: 24 mg/kg per day for 3 days) Formulation: 2.3% MB solution	14 days	Overall clinical and parasitological cure rate on day 14 was 90% (326/364) and 77% (278/364) respectively, without differences between groups	There were three SAEs, one probably associated with MB Haemoglobin development was not associated with G6PD deficiency	MB was given with fruit flavouring and honey supplement to mask the bitter taste
Alving (1949) Cited by Baird et al. USA (2012)^a^ [61]	*n* = 37	*P. vivax*	Clinical	Arm 1: IQ (*n* = 10) Arm 2: IQ-quinine (*n* = 15) Arm 3: IQ-MB (*n* = 9) (MB: 500 mg per day) Arm 4: IQ-MB-quinine (*n* = 3) (MB: 500 mg per day) All treatments were for 14 days	14 days	Arm 1: 9/10 relapsed Arm 2: 5/15 relapsed Arm 3: 3/9 relapsed Arm 4: 0/3 relapsed	1/10 in arm 1 experienced severe haemolysis; after being treated again with IQ plus MB for 14 days, no haemolysis	–
Case series								
Mayer Russia (1919) [62]	*n* = 3	*P. malariae*	By microscope	1000 mg per day over 30 days (16 days MB and 14 days breaks); MB divided into five doses of 200 mg per day	52–72 days	2/3: cure 1/3: relapsed after 4 months	Mild urogenital symptoms despite daily nutmeg application	–
Panse Africa (1902) [63]	*n* = 2	*P. malariae*	By microscope	Case 1: 400–1000 mg per day for 14 days Case 2: 600–1000 mg per day for 32 days	14–32 days	Case 1: cure Case 2: failure	No safety information	Both patients were pretreated with quinine
Glogner Indonesia (1901) [64]	*n* = 6 (2 adults, 4 children)	*P. vivax/ovale*	By microscope	Adults: 1000 mg every 2 days; 1000 mg per day Children: 300 mg per day every 5 days; 300 mg per day every 2 days	2–7 months	6/6 relapsed	No safety information	All patients were pretreated with quinine
Ollwig (1899) Africa [65]	*n* = 10	*P. vivax/ovale* (3/10) *P. falciparum* (4/10) *P. malariae* (1/10) *P. vivax/ovale* and *P. falciparum* (1/10) Unspecified (1/10)	By microscope	300 mg per day to 1000 mg per day for 3 days to 14 days, followed by breaks of 5–8 days. The regimen was cycled up to 3 months	8 days to 3 months	7/10: cured 3/10: failure	Urogenital symptoms (*n* = 2) Vomiting after MB intake (*n* = 3) Diarrhoea (*n* = 1)	Frequent vomiting of MB reported in 2/3 failure cases 6/10 cases were pretreated with quinine Nutmeg was taken together with MB against urogenital symptoms
Cardamatis Greece (1898) [66]	*n* = 275 (157/118 male/female); 129 children, 91 youths, 55 adults	*P. vivax/ovale* (72/275) *P. falciparum* (178/275) *P. malariae* (21/275) Unspecified *(4/275)*	Clinical	In 245/275 MB monotherapy Adults: 400–500 mg per day Youth: 300 mg per day Children: 200 mg per day Infants: 20–40 mg per day Regimens given in four doses per day (every 2 hours) initially for 6–12 days and for a total of 22–60 days (with variable pauses) In 30/275 MB in combination with quinine or arsenic	Up to 1 year	257/275 cured 18/275 failure	Urogenital symptoms observed only with very high MB doses Colouring properties in particular in association with vomiting of children	Good efficacy in quinine non-responders Nutmeg was taken together with MB against urogenital symptoms
Röttger Germany (1895) [67]	*n* = 7	No specific information	Clinical	600–800 mg per day for 8–33 days	8–33 days	6/7 cured 1/7 failure	1/7 vomiting after MB1/7 urogenital symptoms	Nutmeg helped to reduce the urogenital side effects
Ferreira Brazil (1893) [68]	*n* = 21 (2–180-month-old children, median 18 months)	No specific information	Clinical	200–600 mg per day, usually in divided doses, for 3–30 days	3–30 days (median 9 days)	21/21 cured	1/21 reported urogenital symptoms	5/21 initial treatment with quinine failed
Parenski and Blatteis Europe (1893) [69]	*n* = 35	No specific information	By microscope	800–1500 mg per day	7 days to 4 months	33/35 cases cured after 7 days 2/35 failure	Divided small doses (0.1–0.2 g) of MB rarely produced side effectsDivided higher doses (0.4–0.6 g) of MB produced more side effects, in particular vomiting and urogenital symptoms	Medicinale MB Merck free of chlorinated zinc, lead and arsenic was used
Thayer USA (1892) [70]	*n* = 7 (age range: 17–58 years, median 33)	*P. vivax/ovale* (3/7) *P. malariae* (1/7) Unspecified (3/7)	Clinical	400–1000 mg per day for 7–23 days	7–23 days	4/7 cured 3/7 failure	Urogenital symptoms (3/7) Dizziness (1/7)	Nutmeg was taken together with MB to reduce urogenital symptoms
Guttmann and Ehrlich Germany (1891) [29]	*n* = 2	*P. vivax/ovale*	By microscope	500 mg per day for 12–24 days	1–2 months	2/2 cured	Urogenital symptoms (*n* = 1)	Nutmeg was taken together with MB to reduce urogenital symptoms
Case reports								
Mühlens and Kirschbaum Germany (1921) [71]	*n* = 1	*P. vivax/ovale*	By microscope	1000 mg per day for 7 days followed by alternating 5-day breaks and 3-day treatments with 1000 mg per day for 3 months	3 months	Cured	No safety information	Nutmeg was taken together with MB to reduce urogenital symptoms
Atkinson China (1903) [72]	*n* = 1 (male child)	Unspecified	By microscope	300 mg per day	11 days	Cured	Gastrointestinal disorder	Initial treatment with quinine failed
Sivers Germany (1901) cited by Merck (1922) [73, 74]	*n* = 1 (15-year-old girl)	*P. vivax/ovale*	By microscope	500 mg per day	3 days	Cured	Vomiting	–
Anonymous Germany (1893) [75]	*n* = 1 (32-year-old man)	*P. vivax/ovale*	By microscope	1000 mg on day 1, 500 mg on day 2, then 300 mg for 14 days, then continued treatment for 6 weeks	2 months	Parasite-free on day 7, relapse on day 14, and cured without relapse on day 60	Urogenital symptoms	MB dose for treatment after relapse not specified Nutmeg helped to reduce urogenital side effects
Trintignan India (1882) cited by Röttger (1895) [67]	*n* = 1	*P. falciparum*	Clinical	2000 mg per day during acute attack, followed by 500 mg per day until day 20	20 days	Cured	None	–

*ACPR* adequate clinical and parasitological response, *AQ* amodiaquine, *AS* artesunate, *CQ* chloroquine, *IQ* isopentaquine, *MB* methylene blue, *SAE* serious adverse event

^a^Controlled phase II study

^b^Phase II dose-finding study

### Study designs and MB regimens

Fifteen studies were historical case reports (*n* = 5) or case series (*n* = 10), while six studies were non-randomised controlled trials (*n* = 3) or RCTs (*n* = 3) from more recent years.

Summaries of treatment regimens are shown in Table 1. In the historical studies, MB was usually given in divided doses (e.g. five times per day) of 300–1000 mg per day in adults and 20–300 mg per day in children for 3–90 days as daily or interrupted (e.g. a 2-day pause after several days of treatment) oral monotherapy, with largely varying follow-up periods [29, 62, 64–67, 69]. In the controlled study on vivax malaria, MB was given to adults at a dose of 500 mg per day for 14 days in combination with isopentaquine (IQ) or in combination with IQ and quinine (Q) [61]. In the very recent RCTs on falciparum malaria, MB was given to children in various combination regimens [MB- CQ, MB-amodiaquine (AQ), MB-artesunate (AS) or MB-AQ-AS] in doses ranging from 4 mg/kg per day to 24 mg/kg per day, initially as 2–4 divided doses per day, and always for 3 days [56, 58, 60]. The latest dose regimen applied was 15 mg/kg per day in a single dose over 3 days [55]. In the only recently conducted MB monotherapy study, MB in adults was administrated at a fixed dose of 780 mg per day for 3, 5 or 7 days [59]. In the controlled studies, comparator regimens were CQ, AS-AQ, IQ and IQ-Q [56, 58, 60, 61].

### Quality of included studies

Tables 2, 3 and 4 show the quality assessment of the studies included. The three RCTs had a low risk for bias and were consequently considered to be of high quality. Of the three non-RCTs, the two recently conducted studies scored rather high and were, thus, considered of high quality, while the study from 1949 had a relatively low quality score. The quality of the case series and case reports finally included in our review was overall high. The mean quality score was 10.53, and the majority of case series and case reports provided a clear description of malaria treatment and efficacy outcomes. The information most frequently missing from case series and reports was study participant characteristics (33.3%, 5/15) and safety outcomes (26.7%, 4/15).

**Table 2:** Quality assessment of included randomised controlled trials

Study: First Author Place of study (Year)	Selection bias^a^ (random sequence generation)		Performance bias^a^ (blinding of the participants and personnel)	Detection bias^a^ (blinding of outcome assessment)	Attribution bias^a^ (incomplete outcome data)	Reporting bias^a^(selective reporting)
	Allocation concealment bias^a^					
Coulibaly et al*.* Burkina Faso (2015) [55]	Low	Low	Low	Low	Low	Low
Zoungrana et al. Burkina Faso (2008) [56, 57]	Low	Low	Low	Low	Low	Low
Meissner et al. Burkina Faso (2005) [58]	Low	Low	Low	Low	Low	Low

^a^Assessed as low, unclear or high risk of bias

**Table 3:** Quality assessment of included non-randomised controlled trials

Methodological item	Score for studies^a^		
	Bountogo et al. Burkina Faso (2010) [59]	Meissner et al. Burkina Faso (2006) [60]	Alving (1949) Cited by Baird et al. (2012) [61]
Clearly stated aim	2	2	1
Inclusion of consecutive patients	2	2	1
Prospective collection of data	2	2	0
End points appropriate to the aim of the study	1	2	1
Unbiased assessment of the study end point	2	2	0
Follow-up period appropriate to the aim of the study	2	2	1
Loss to follow-up less than 5%	2	2	0
Prospective calculation of the study size	2	2	0
Adequate control group	1	2	1
Contemporary groups	1	1	1
Baseline equivalence of groups	1	2	0
Adequate statistical analyses	2	2	0
Total score	20	23	6

^a^The items are scored 0 (not reported), 1 (reported but inadequate) or 2 (reported and adequate). The global ideal score was 24 for comparative studies

**Table 4:** Quality assessment of included case series and case reports

Study: First Author Place of study (Year)	Score for studies^a^							Total score
	What is the overall degree of presentation of study details?	Detailed presentation of study participant characteristics?	Case definition clearly reported?	Clear description of malaria treatment reported?	Clear reporting on the follow-up period?	Appropriate reporting of efficacy outcomes?	Appropriate reporting of safety outcomes?	
Case series								
Mayer Russia (1919) [62]	2	0	2	2	2	2	2	12
Panse Africa (1902) [63]	2	0	2	2	1	1	0	8
Glogner Indonesia (1901) [64]	2	2	2	2	1	1	0	10
Ollwig Africa (1899) [65]	2	1	1	2	1	2	2	11
Cardamatis Greece (1898) [66]	1	0	1	2	1	1	1	7
Röttger Germany (1895) [67]	2	2	1	2	1	2	2	12
Ferreira Brazil (1893) [68]	2	2	1	2	2	2	2	13
Parenski and Blatteis Europe (1893) [69]	1	0	1	2	1	1	1	7
Thayer USA (1892) [70]	2	2	1	2	1	2	2	12
Guttmann and Ehrlich Germany (1891) [29]	2	2	2	2	1	2	2	13
Case reports								
Mühlens Germany (1921) [71]	1	0	2	2	2	2	0	9
Atkinson China (1903) [72]	1	1	1	2	2	2	1	10
Sivers Germany (1901) cited by Merck (1922) [73, 74]	1	2	2	2	1	1	2	11
Anonymous Germany (1893) [75]	2	2	2	2	2	2	2	14
Trintignan India (1882) cited by Röttger (1895) [67]	1	1	1	2	2	2	0	9

^a^The items are scored 0 (not reported), 1 (reported but inadequate) or 2 (reported and adequate). The global ideal score was 14 for case series and case reports

### Efficacy of MB against asexual parasites

In MB monotherapy studies conducted around the year 1900, 337/373 (90%) of malaria cases were reported to be cured. Reported potential reasons for treatment failure were: unsuccessful quinine pretreatment (*n* = 8), vomiting of treatment (*n* = 3), low dose of MB (*n* = 6) and unknown (*n* = 19). In a more recent trial, MB monotherapy in 60 West African adults with falciparum malaria (a fixed dose of 780 mg for 3 days) showed a 100% cure rate when MB was given for 7 days compared to a 85% cure rate when MB was given for a shorter time period; this difference was close to being statistically significant [59].

Like MB in the combination treatment, a controlled study on vivax malaria among US prisoners from 1949 reported a cure rate of 6/9 (67%) for MB plus IQ and of 3/3 (100%) for MB plus IQ plus quinine [61]. In the recent RCTs among West African children with falciparum malaria, MB-based regimens (*n* = 391) showed superior efficacy compared to control regimens (*n* = 207). In the first trial, the cure rate of MB (4 mg/kg per day) plus CQ (56%) was slightly but non-significantly higher compared to that of CQ alone (46%) [58]. A subsequent dose-finding study demonstrated an improved efficacy (77%) of MB-CQ when higher doses of MB (12–24 mg/kg per day) were used [60]. A third study compared the regimens MB-AQ, MB-AS (MB at 20 mg/kg per day) and AS-AQ. The cure rates were 95%, 62% and 82%, respectively, and these differences were statistically significant [56]. Finally, a trial that compared AS-AQ-MB (MB at 15 mg/kg per day) with AS-AQ produced similar cure rates in both regimens (80% vs 85%) [55].

Parasite clearance with MB appeared to be rather slow. In African adults treated with MB monotherapy, 9% were still parasitaemic on day 3 [59]. In children treated with MB-CQ or CQ, the median parasite clearance time did not differ (91.3 and 86.4 h) [58]. In children treated with MB-AS, only 2% were parasitaemic on day 3, i.e., a significantly lower proportion than following treatment with AS-AQ (5%) or MB-AQ (17%) [56]. The clearance time for the *P. falciparum* parasite was non-significantly shortened in children receiving AS-AQ-MB compared to the AS-AQ group (medians 43.7 vs 47.0 h) [55]. Fever was cleared rapidly in all of these studies without differences between MB and comparator regimens [55, 56, 58].

### Efficacy of MB against gametocytes

Gametocyte clearance following MB-based treatment was investigated in two RCTs. The first study was a secondary analysis of data from an RCT in children. Compared to AS-AQ, both MB-containing regimens were associated with significantly reduced gametocyte carrier rates during follow-up days 3, 7 and 14 [57]. The second study, in which post-treatment gametocyte prevalence was the main outcome variable, demonstrated a significantly lower figure in children treated with MB-AS-AQ compared to AS-AQ on day 7 of follow-up (microscopically, 1% vs 9%; by QT-NASBA, 37% vs 63%) [55].

### Safety and acceptability of MB

Treatment of malaria with MB was consistently associated with green-blue discoloration of urine. AEs concerning the urogenital system (urethritis) and the gastrointestinal system (vomiting) were reported to be associated with MB treatment. These AEs were more frequently reported with higher doses of MB [65, 66, 69, 75]. Vomiting in children was often associated with the bitter taste of MB, depending on the type of formulation. In two RCTs, vomiting in MB-containing regimens ranged from 24% to 68% [55, 56]. Urethritis was reported in 78% of adults and in 55% of older children treated with MB, but this symptom was not reported from RCTs in preschool children [56, 59]. In the controlled studies, severe adverse events (SAEs) were rarely reported and none were attributed to MB [60]. In particular, there were no cases of severe haemolysis associated with MB.

Despite some AEs being clearly associated with MB treatment, one study reported no differences between study groups in acceptance rates by parents and caregivers of children [55].

## Discussion

This is the first review within the last 100 years to attempt systematically to collect and analyse all the data that have been published on the effects of MB in the treatment of human malaria. As MB was used for this indication globally in the late 19th and early 20th centuries, some early reviews were published at that time. In 1904, a meta-analysis of 425 malaria cases from 11 publications concluded that MB was effective in 85% of patients [30].

In the early 20th century, MB was gradually replaced by new synthetic antimalarials with different characteristics and finally without colouring properties [9]. Scientists at Bayer used MB as the starting point for systematic antimalarial testing of synthetic compounds. Thus, the first synthetic drug designed as an antimalarial, pamaquine, was derived from MB [76].

The revival of MB as an antimalarial drug candidate began in 1995, at the height of the development of resistance against existing antimalarials, in three biochemical laboratories [32, 35, 38]. A key achievement was the detection that MB inhibits the glutathione reductase of *P. falciparum* [8]. Considerably elevated glutathione levels were found in CQ-resistant *P. falciparum* strains, triggering the hypothesis that combining MB and CQ might overcome resistance [8]. This was the rationale to start clinical trials with MB in the early 21st century.

### Efficacy of MB against malaria

In our analysis of 15 case reports and case series, 339/373 (91%) of patients were cured, which provides evidence for the high efficacy of MB monotherapy in the treatment of malaria. Treatment failures were attributed to previously unsuccessful quinine treatment, vomiting and a low and/or short MB dosing regimen, which helps to explain early controversies on whether quinine or MB was more effective against malaria [63–65, 77]. Conducted in tropical as well as in non-tropical areas and including all types of human malaria, these early studies were of overall good external validity. Interestingly, the MB dosing schedules used in these early studies were rather similar to what has been found to be effective in a MB dose-finding study in African children as well as in a proof of principle MB monotherapy study in African adults [59, 60].

In the recent studies, MB was usually given in combination with other antimalarials and tested in RCTs. In an area of high CQ resistance, MB-CQ was more effective than CQ alone but not sufficiently so [58, 60]. Combining MB with AS, AQ or AS-AQ and increasing MB doses in these combinations did not lead to a substantial increase in efficacy [55, 56]. This indicates that the curative efficacy of MB in eliminating asexual parasitaemia appears to be limited in the study region of Burkina Faso compared to the cure rates observed roughly a hundred years ago in a variety of settings. There is no evidence for and—because of the lack of drug pressure—no reason to expect that MB resistance has emerged meanwhile. The actual causes of the comparatively lower efficacy of MB-containing regimens in the recent studies, thus, remains unclear. Total dosage and a generally longer treatment period in the older studies might be relevant, however.

Despite this limitation, other properties of MB are notable and promising. Adding MB to an ACT reinforced the particular beneficial effects of the artemisinins, i.e., it further accelerated the elimination of asexual *P. falciparum* parasites and reduced *P. falciparum* gametocytes [55–57]. The epidemiological relevance of the latter observation has recently been confirmed by a phase II study in Mali, which showed a 100% reduction of mosquito infectivity by day 7 both with PQ- and MB-containing drug regimens through membrane feeding assays [78]. This supports findings from preclinical studies on an existing synergy between MB and artemisinins as well as on the very strong effects of MB on *P. falciparum* gametocyte reduction [41, 44, 45, 79]. Interestingly, the efficacy of MB against gametocytes had been observed in historical studies [30]. However, further studies to identify the lowest effective dose for the gametocytocidal effect of MB in falciparum malaria should be conducted.

### Safety of MB in malaria treatment

In the historical studies, MB was usually given orally and often in high doses and for prolonged periods of time, both in children and in adults, and without reports of major safety problems. During World War I for example, some European soldiers received more than 400 g of MB over several weeks without major side effects, apart from moderate urogenital symptoms [31]. Brazilian children were reported to tolerate 20–50 mg/kg per day of MB very well for long periods of time [68]. Also, in the recent RCTs conducted in West Africa, MB treatment was not associated with SAEs. However, while MB given orally seems to be largely well tolerated, MB given intravenously must be applied with caution. Intravenous MB is often routinely given as the first-line treatment for acute acquired methemoglobinemia in doses of 1–2 mg/kg, but a dose of 7 mg/kg can lead to severe gastrointestinal symptoms [9]. Moreover, a dose of 5 mg/kg has been reported to be associated with an altered mental status during parathyroidectomy [80]. For sheep, the LD50 of MB was found to be 42 mg/kg when applied intravenously [81].

AEs shown to be associated with MB treatment are mild gastrointestinal symptoms, which may manifest as vomiting, and mild urogenital symptoms, which usually manifest shortly after drug intake. This has consistently been reported, both in historical studies and in more recently conducted RCTs. Pure MB powder or MB dissolved in water has a very bitter and metallic taste. The gastrointestinal symptoms are clearly influenced by the formulation of MB, which suggests it is better to use taste-masked formulations. As the World Health Organization is now also recommending solid formulations for small children, mini-tablets, which should also be taste-masked, will be preferred over liquid formulations [82]. MB given with food and with a small amount of grated nutmeg has consistently been described as being effective in suppressing the frequently reported gastrointestinal and urogenital AEs [30, 60, 66]. Finally, both MB and PQ were recently shown to be well tolerated in males in Mali [78].

MB is on the list of drugs potentially dangerous for patients with G6PD deficiency, but the clinical importance of this is still controversial [83, 84]. In this review, no association between MB and severe haemolysis has been detected. Nevertheless, in a pooled analysis of all recent studies conducted with MB against falciparum malaria in West African children (including one unpublished RCT), small effects were seen. In 844 MB-treated African children, two patients developed severe anaemia (Hb < 5 g/dL) during the first days of treatment and both were G6PD deficient. Minimal Hb concentrations following MB treatment did not significantly differ in children with and without G6PD deficiency. However, when modelling the Hb time course, an MB dose-dependent effect of lowering Hb concentrations was observed for children with a full G6PD defect (hemi- and homozygous deficiency; prevalence, 10%). The maximal difference compared to non-deficient peers was estimated as −0.9 g/dl on day 5 of the follow-up, which is, however, considered to be of limited clinical relevance [85]. In sub-Saharan Africa, the moderate A minus type of X-chromosomally inherited G6PD deficiency (15–25% enzyme activity) dominates and affects 10–25% of the population. G6PD-deficient red blood cells have increased sensitivity to oxidative stress originating from various triggers including antimalarial drugs [86]. Whether or not MB confers an increased risk in malaria patients with more severe variants of G6PD deficiency needs to be evaluated in future studies, e.g. in South-East Asia. There are, however, reports of MB being associated with severe haemolytic reactions in neonatal G6PD deficiency [87, 88]. Finally, MB at low concentration is a strong inhibitor of monoamine oxidase A and therefore, should not be given together with serotonin reuptake inhibitors [89].

Reassuringly and despite the characteristic AE profile, MB has been well accepted by patients and caregivers of children in West Africa, which was also supported by an anthropological study in Burkina Faso [90]. However, the acceptance of its colouring properties also needs to be studied in other populations and cultures.

### Strengths and limitations

The strength of this review is that it includes all studies ever conducted with MB or MB-containing regimens in humans with malaria irrespective of study design and sample size and irrespective of the language of the report. Moreover, it can be considered as beneficial that a number of authors of this review have been the investigators of all recently conducted studies on this topic. Limitations of this review are the lower quality of the historical studies included compared to recent RCTs, the availability of three reports only in an abbreviated form from other sources [73, 91, 92] and the non-availability of a number of studies due to their old age despite extensive searches.

## Conclusions

This review shows that MB has substantial antimalarial activity against all types of malaria in various endemic areas and, in combination with other antimalarials, against falciparum malaria in Africa. Although MB alone appears to act rather slowly against the asexual parasites of *P. falciparum*, it shows synergy with the artemisinin component in rapidly clearing the parasites and it is very effective in reducing the gametocytes and consequently mosquito transmission. MB is usually well tolerated and accepted with mild and regularly self-limiting gastrointestinal and urogenital symptoms, which are the main AEs. To avoid vomiting, the drug should be given in a taste-masked formulation and/or together with food. In historical studies, nutmeg has been shown to be effective in moderating the frequent occurrence of urethritis, which should be evaluated in future studies. Whether the small effect of MB on the haemoglobin development is of clinical significance needs to be monitored in future large-scale studies including regions with more severe forms of G6PD deficiency compared to African populations.

MB appears to be a potential alternative to PQ for reducing post-treatment infectivity in *P. falciparum* infections, a useful partner for triple combination therapy regimens with the goal of protecting the artemisinin component of the ACT and of reducing the spread of drug-resistant parasites, and a potentially valuable partner drug for mass drug administration in malaria elimination programs. Further studies should investigate the efficacy, safety and community acceptance of different ACT regimens in combination with MB against falciparum malaria in different areas inside and outside Africa, while well-designed pilot studies should investigate the effects of MB and MB-containing combination regimens as a treatment for vivax malaria.

## Additional files

Additional file 1: Search strategy. (DOCX 12 kb)

Additional file 2: A list of all the records screened after duplicates were removed (*N* = 474). (PDF 329 kb)

## Abbreviations

ACT: Artemisinin-based combination therapy
AE: Adverse event
AQ: Amodiaquine
AS: Artesunate
CQ: Chloroquine
IQ: Isopentaquine
MB: Methylene blue
PQ: Primaquine
Q: Quinine
RCT: Randomised controlled trial
SAE: Severe adverse event

## References

[1] White NJ, Pukrittayakamee S, Hien TT, Faiz MA, Mokuolu OA, Dondorp AM (2014). Malaria. Lancet.

[2] Nosten F, Brasseur P (2002). Combination therapy for malaria: the way forward?. Drugs.

[3] Adjuik M, Babiker A, Garner P, Olliaro P, Taylor W, White N, International Artemisinin Study G (2004). Artesunate combinations for treatment of malaria: meta-analysis. Lancet.

[4] WHO. World malaria report 2016. Geneva: World Health Organization; 2016

[5] Noedl H, Se Y, Schaecher K, Smith BL, Socheat D, Fukuda MM, Artemisinin Resistance in Cambodia 1 Study C (2008). Evidence of artemisinin-resistant malaria in western Cambodia. N Engl J Med.

[6] White NJ (2004). Antimalarial drug resistance. J Clin Invest.

[7] WHO. Guidelines for the treatment of malaria-3rd edition. Geneva: World Health Organization; 2015. 26020088

[8] Schirmer RH, Coulibaly B, Stich A, Scheiwein M, Merkle H, Eubel J, Becker K, Becher H, Muller O, Zich T (2003). Methylene blue as an antimalarial agent. Redox Rep.

[9] Schirmer RH, Adler H, Pickhardt M, Mandelkow E (2011). Lest we forget you--methylene blue. Neurobiol Aging.

[10] Wainwright M, Amaral L (2005). The phenothiazinium chromophore and the evolution of antimalarial drugs. Tropical Med Int Health.

[11] Warth A, Goeppert B, Bopp C, Schirmacher P, Flechtenmacher C, Burhenne J (2009). Turquoise to dark green organs at autopsy. Virchows Arch.

[12] Walter-Sack I, Rengelshausen J, Oberwittler H, Burhenne J, Mueller O, Meissner P, Mikus G (2009). High absolute bioavailability of methylene blue given as an aqueous oral formulation. Eur J Clin Pharmacol.

[13] DiSanto AR, Wagner JG (1972). Pharmacokinetics of highly ionized drugs. II. Methylene blue--absorption, metabolism, and excretion in man and dog after oral administration. J Pharm Sci.

[14] Peter C, Hongwan D, Kupfer A, Lauterburg BH (2000). Pharmacokinetics and organ distribution of intravenous and oral methylene blue. Eur J Clin Pharmacol.

[15] Mansouri A, Lurie AA (1993). Concise review: methemoglobinemia. Am J Hematol.

[16] Coleman MD, Coleman NA (1996). Drug-induced methaemoglobinaemia. Treatment issues. Drug Saf.

[17] Cawein M, Behlen CH, Lappat EJ, Cohn JE (1964). Hereditary Diaphorase Deficiency and Methemoglobinemia. Arch Intern Med.

[18] Zulian GB, Tullen E, Maton B (1995). Methylene blue for ifosfamide-associated encephalopathy. N Engl J Med.

[19] Kupfer A, Aeschlimann C, Wermuth B, Cerny T (1994). Prophylaxis and reversal of ifosfamide encephalopathy with methylene-blue. Lancet.

[20] Orth K, Ruck A, Stanescu A, Beger HG (1995). Intraluminal treatment of inoperable oesophageal tumours by intralesional photodynamic therapy with methylene blue. Lancet.

[21] O'Leary JL, Petty J, Harris AB, Inukai J (1968). Supravital staining of mammalian brain with intra-arterial methylene blue followed by pressurized oxygen. Stain Technol.

[22] Williamson LM, Cardigan R, Prowse CV (2003). Methylene blue-treated fresh-frozen plasma: what is its contribution to blood safety?. Transfusion.

[23] Floyd RA, Schneider JE, Dittmer DP (2004). Methylene blue photoinactivation of RNA viruses. Antivir Res.

[24] Van der Horst C, Stuebinger H, Seif C, Melchior D, Martinez-Portillo FJ, Juenemann KP (2003). Priapism - etiology, pathophysiology and management. Int Braz J Urol.

[25] Juffermans NP, Vervloet MG, Daemen-Gubbels CRG, Binnekade JM, de Jong M, Groeneveld ABJ (2010). A dose-finding study of methylene blue to inhibit nitric oxide actions in the hemodynamics of human septic shock. Nitric Oxide-Biol Ch.

[26] Levin RL, Degrange MA, Bruno GF, Del Mazo CD, Taborda DJ, Griotti JJ, Boullon FJ (2004). Methylene blue reduces mortality and morbidity in vasoplegic patients after cardiac surgery. Ann Thorac Surg.

[27] Oz M, Lorke DE, Petroianu GA (2009). Methylene blue and Alzheimer's disease. Biochem Pharmacol.

[28] Wischik CM, Edwards PC, Lai RY, Roth M, Harrington CR (1996). Selective inhibition of Alzheimer disease-like tau aggregation by phenothiazines. Proc Natl Acad Sci U S A.

[29] Guttmann P, Ehrlich P (1891). Über die wirkung des methylenblau bei malaria. Berliner Klin Wochenschr.

[30] Wood HC (1904). The use of methylene blue in malarial fevers. Proc Phila Co Med Soc.

[31] Marshall DG (1920). The "toxicity" of methylene-blue. Lancet.

[32] Farber PM, Arscott LD, Williams CH, Becker K, Schirmer RH (1998). Recombinant Plasmodium falciparum glutathione reductase is inhibited by the antimalarial dye methylene blue. FEBS Lett.

[33] Sarma GN, Savvides SN, Becker K, Schirmer M, Schirmer RH, Karplus PA (2003). Glutathione reductase of the malarial parasite Plasmodium falciparum: crystal structure and inhibitor development. J Mol Biol.

[34] Buchholz K, Schirmer RH, Eubel JK, Akoachere MB, Dandekar T, Becker K, Gromer S (2008). Interactions of methylene blue with human disulfide reductases and their orthologues from Plasmodium falciparum. Antimicrob Agents Chemother.

[35] Pastrana-Mena R, Dinglasan RR, Franke-Fayard B, Vega-Rodriguez J, Fuentes-Caraballo M, Baerga-Ortiz A, Coppens I, Jacobs-Lorena M, Janse CJ, Serrano AE (2010). Glutathione reductase-null malaria parasites have normal blood stage growth but arrest during development in the mosquito. J Biol Chem.

[36] Atamna H, Krugliak M, Shalmiev G, Deharo E, Pescarmona G, Ginsburg H (1996). Mode of antimalarial effect of methylene blue and some of its analogues on Plasmodium falciparum in culture and their inhibition of P. vinckei petteri and P. yoelii nigeriensis in vivo. Biochem Pharmacol.

[37] Kanzok SM, Schirmer RH, Turbachova I, Iozef R, Becker K (2000). The thioredoxin system of the malaria parasite Plasmodium falciparum. Glutathione reduction revisited. J Biol Chem.

[38] Davioud-Charvet E, Delarue S, Biot C, Schwobel B, Boehme CC, Mussigbrodt A, Maes L, Sergheraert C, Grellier P, Schirmer RH (2001). A prodrug form of a Plasmodium falciparum glutathione reductase inhibitor conjugated with a 4-anilinoquinoline. J Med Chem.

[39] Thurston JP (1953). The chemotherapy of Plasmodium berghei. I. Resistance to drugs. Parasitology.

[40] Vennerstrom JL, Makler MT, Angerhofer CK, Williams JA (1995). Antimalarial dyes revisited: xanthenes, azines, oxazines, and thiazines. Antimicrob Agents Chemother.

[41] Ohrt C, Li Q, Obaldia N, Im-Erbsin R, Xie L, Berman J (2014). Efficacy of intravenous methylene blue, intravenous artesunate, and their combination in preclinical models of malaria. Malar J.

[42] Pascual A, Henry M, Briolant S, Charras S, Baret E, Amalvict R, Huyghues d, Etages E, Feraud M, Rogier C, Pradines B (2011). In vitro activity of Proveblue (methylene blue) on *Plasmodium falciparum* strains resistant to standard antimalarial drugs. Antimicrob Agents Chemother.

[43] Wirjanata G, Sebayang BF, Chalfein F, Prayoga, Handayuni I, Trianty L, Kenangalem E, Noviyanti R, Campo B, Poespoprodjo JR (2015). Potent Ex Vivo Activity of Naphthoquine and Methylene Blue against Drug-Resistant Clinical Isolates of Plasmodium falciparum and Plasmodium vivax. Antimicrob Agents Chemother.

[44] Akoachere M, Buchholz K, Fischer E, Burhenne J, Haefeli WE, Schirmer RH, Becker K (2005). In vitro assessment of methylene blue on chloroquine-sensitive and -resistant Plasmodium falciparum strains reveals synergistic action with artemisinins. Antimicrob Agents Chemother.

[45] Adjalley SH, Johnston GL, Li T, Eastman RT, Ekland EH, Eappen AG, Richman A, Sim BK, Lee MC, Hoffman SL (2011). Quantitative assessment of Plasmodium falciparum sexual development reveals potent transmission-blocking activity by methylene blue. Proc Natl Acad Sci U S A.

[46] Garavito G, Bertani S, Rincon J, Maurel S, Monje MC, Landau I, Valentin A, Deharo E (2007). Blood schizontocidal activity of methylene blue in combination with antimalarials against Plasmodium falciparum. Parasite.

[47] Haynes RK, Cheu KW, Li KY, Tang MM, Wong HN, Chen MJ, Guo ZF, Guo ZH, Coghi P, Monti D (2011). A partial convergence in action of methylene blue and artemisinins: antagonism with chloroquine, a reversal with verapamil, and an insight into the antimalarial activity of chloroquine. ChemMedChem.

[48] Garavito G, Bertani S, Quiliano M, Valentin A, Aldana I, Deharo E (2012). The in vivo antimalarial activity of methylene blue combined with pyrimethamine, chloroquine and quinine. Mem Inst Oswaldo Cruz.

[49] Müller O, Sie A, Meissner P, Schirmer RH, Kouyate B (2009). Artemisinin resistance on the Thai-Cambodian border. Lancet.

[50] Liberati A, Altman DG, Tetzlaff J, Mulrow C, Gotzsche PC, Ioannidis JP, Clarke M, Devereaux PJ, Kleijnen J, Moher D (2009). The PRISMA statement for reporting systematic reviews and meta-analyses of studies that evaluate health care interventions: explanation and elaboration. PLoS Med.

[51] Higgins JP, Altman DG, Gotzsche PC, Juni P, Moher D, Oxman AD, Savovic J, Schulz KF, Weeks L, Sterne JA (2011). The Cochrane Collaboration's tool for assessing risk of bias in randomised trials. BMJ.

[52] Slim K, Nini E, Forestier D, Kwiatkowski F, Panis Y, Chipponi J (2003). Methodological index for non-randomized studies (minors): development and validation of a new instrument. ANZ J Surg.

[53] Critical appraisal tools-checklist for case reports. http://joannabriggs.org/assets/docs/critical-appraisal-tools/JBI_Critical_Appraisal-Checklist_for_Case_Reports2017.pdf.

[54] Critical appraisal tools-checklist for case series. http://joannabriggs.org/assets/docs/critical-appraisal-tools/JBI_Critical_Appraisal-Checklist_for_Case_Series2017.pdf.

[55] Coulibaly B, Pritsch M, Bountogo M, Meissner PE, Nebie E, Klose C, Kieser M, Berens-Riha N, Wieser A, Sirima SB (2015). Efficacy and safety of triple combination therapy with artesunate-amodiaquine-methylene blue for falciparum malaria in children: a randomized controlled trial in Burkina Faso. J Infect Dis.

[56] Zoungrana A, Coulibaly B, Sie A, Walter-Sack I, Mockenhaupt FP, Kouyate B, Schirmer RH, Klose C, Mansmann U, Meissner P (2008). Safety and efficacy of methylene blue combined with artesunate or amodiaquine for uncomplicated falciparum malaria: a randomized controlled trial from Burkina Faso. PLoS One.

[57] Coulibaly B, Zoungrana A, Mockenhaupt FP, Schirmer RH, Klose C, Mansmann U, Meissner PE, Muller O (2009). Strong gametocytocidal effect of methylene blue-based combination therapy against falciparum malaria: a randomised controlled trial. PLoS One.

[58] Meissner PE, Mandi G, Witte S, Coulibaly B, Mansmann U, Rengelshausen J, Schiek W, Jahn A, Sanon M, Tapsoba T (2005). Safety of the methylene blue plus chloroquine combination in the treatment of uncomplicated falciparum malaria in young children of Burkina Faso [ISRCTN27290841]. Malar J.

[59] Bountogo M, Zoungrana A, Coulibaly B, Klose C, Mansmann U, Mockenhaupt FP, Burhenne J, Mikus G, Walter-Sack I, Schirmer RH (2010). Efficacy of methylene blue monotherapy in semi-immune adults with uncomplicated falciparum malaria: a controlled trial in Burkina Faso. Tropical Med Int Health.

[60] Meissner PE, Mandi G, Coulibaly B, Witte S, Tapsoba T, Mansmann U, Rengelshausen J, Schiek W, Jahn A, Walter-Sack I (2006). Methylene blue for malaria in Africa: results from a dose-finding study in combination with chloroquine. Malar J.

[61] Baird KJ, Maguire JD, Price RN (2012). Diagnosis and treatment of Plasmodium vivax malaria. Adv Parasitol.

[62] Mayer M (1919). Über die Wirkung von Methylenblau bei Malaria quartana. Deutsch Med Wochenschrift.

[63] Panse O (1902). Schwarzwasserfieber. Aus dem Gouvernementskrankenhaus Taga, Deutsch-Ostafrika.

[64] Glogner M (1900). Ein Beitrag zur Beurtheilung der Malaria Rezidive und ihrer Behandlung.

[65] Ollwig D (1899). Ein Beitrag zur Behandlung der Malaria mit Methylenblau. Z Hyg.

[66] Cardamatis JP (1898). 275 klinische Beobachtungen über das Methylenblau. Deutsche Med Wochenschrift Ther Beilage.

[67] Röttger W (1895). Ein Beitrag zur Behandlung der Malaria mit Methylenblau.

[68] Ferreira MC (1893). Sur l'emploi du bleu de méthylène dans la malaria infantile. Ther Medico-Chirugicale.

[69] Parenski S, Blatteis S (1893). Über das Methylenblau bei Malaria. Ther Monatshefte.

[70] Thayer WS (1892). On the Value of methylene blue in malarial fever. Bull Johns Hopkins Hosp.

[71] Mühlens P, Kirschbaum W (1921). Parasitologische und klinische Beobachtungen bei künstlichen Malaria und Recurrensübertragungen. Z Hyg Infekt.

[72] Atkinson JM (1903). Ocimum Viride and Malaria. Lancet.

[73] Sivers R (1901). Finska Läkaresällskapets Handlingar.

[74] Merck E (1922). Merck's Wissenschaftliche Abhandlungen aus den Gebieten der Pharmakotherapie, Pharmazie und verwandter Disziplinen-Anilinfarben in der Therapie.

[75] Anonymous (1893). Über Methylenblau. Dtsch Med Wochenschr.

[76] Krafts K, Hempelmann E, Skorska-Stania A (2012). From methylene blue to chloroquine: a brief review of the development of an antimalarial therapy. Parasitol Res.

[77] Kaufmann P (1919). Ueber die Wirkung des Methylenblau bei Malaria. Dtsch Med Wochenschr.

[78] Dicko A, Roh ME, Diawara H, Mahamar A, Soumare HM, Lanke K, Bradley J, Sanogo K, Kone DT, Diarra K, et al. Efficacy and safety of primaquine and methylene blue for prevention of Plasmodium falciparum transmission in Mali: a phase 2, single-blind, randomised controlled trial. Lancet Infect Dis. 2018. 10.1016/S1473-3099(18)30044-6. Epub ahead of print. 10.1016/S1473-3099(18)30044-6 PMC5968371 29422384

[79] Siciliano G, Santha Kumar TR, Bona R, Camarda G, Calabretta MM, Cevenini L, Davioud-Charvet E, Becker K, Cara A, Fidock DA (2017). A high susceptibility to redox imbalance of the transmissible stages of Plasmodium falciparum revealed with a luciferase-based mature gametocyte assay. Mol Microbiol.

[80] Kahn M, North A, Chadwick D (2007). Prolonged postoperative altered mental status after methylene blue infusion during parathyroidectomy: a case report and review of literature. Ann R Coll Surg Engl.

[81] Burrows GE (1984). Methylene blue: effects and disposition in sheep. J Vet Pharmacol Ther.

[82] Orubu ES, Tuleu C (2017). Medicines for children: flexible solid oral formulations. Bull World Health Organ.

[83] Youngster I, Arcavi L, Schechmaster R, Akayzen Y, Popliski H, Shimonov J, Beig S, Berkovitch M (2010). Medications and glucose-6-phosphate dehydrogenase deficiency: an evidence-based review. Drug Saf.

[84] Müller O, Meissner P, Mansmann U (2012). Glucose-6-phosphate dehydrogenase deficiency and safety of methylene blue. Drug Saf.

[85] Müller O, Mockenhaupt FP, Marks B, Meissner P, Coulibaly B, Kuhnert R, Buchner H, Schirmer RH, Walter-Sack I, Sie A (2013). Haemolysis risk in methylene blue treatment of G6PD-sufficient and G6PD-deficient West-African children with uncomplicated falciparum malaria: a synopsis of four RCTs. Pharmacoepidem Dr S.

[86] Cappellini MD, Fiorelli G (2008). Glucose-6-phosphate dehydrogenase deficiency. Lancet.

[87] Gauthier TW (2000). Methylene blue-induced hyperbilirubinemia in neonatal glucose-6-phosphate dehydrogenase (G6PD) deficiency. J Matern Fetal Med.

[88] Kirsch I, Cohen H (1980). Heinz body hemlytic anemia from the use of methylene blue in neonates. J Pediatr.

[89] Ramsay RR, Dunford C, Gillman PK (2007). Methylene blue and serotonin toxicity: inhibition of monoamine oxidase A (MAO A) confirms a theoretical prediction. Br J Pharmacol.

[90] Sanon S, Ollivier E, Azas N, Mahiou V, Gasquet M, Ouattara CT, Nebie I, Traore AS, Esposito F, Balansard G (2003). Ethnobotanical survey and in vitro antiplasmodial activity of plants used in traditional medicine in Burkina Faso. J Ethnopharmacol.

[91] Trintignan (1892). Du bleu de methylene dans le paludisme et dans la blenhorragie. Bull Med Paris.

[92] Alving AS, Eichelberger L, Arnold JJE. The clinical testing of antimalarial drugs at Stateville Penitentiary. Semi-Annual Report 1948–49 1949, NIH Malaria Report NO. 87. (USPHS Antimalarial Grant No.RG 198).

