# Supplementary material for: Efficacy and safety of methylene blue in the treatment of malaria: a systematic review
Source: BMC Med. 2018 Apr 25;16:59. doi: 10.1186/s12916-018-1045-3 (PMC5979000; doi:10.1186/s12916-018-1045-3)
Supplement: Supplementary file 1 — Search strategy. (DOCX 12 kb) [file 12916_2018_1045_MOESM1_ESM.docx]

**Supplemental material:**

**MEDLINE search strategy:**

1. malaria

2. vivax

3. falciparum

4. blackwater fever

5. (1 OR 2 OR 3 OR 4)

6. methylene blue

7. methylenblau

8. (6 OR 7)

9. 5 AND 8

**Search Strategy:**

(("malaria"[MeSH Terms] OR "malaria"[All Fields]) OR ("Malaria"[Mesh] OR "Malaria, Vivax"[Mesh] OR "Malaria, Falciparum"[Mesh] OR "Blackwater Fever"[Mesh])) AND (("methylene blue"[MeSH Terms] OR ("methylene"[All Fields] AND "blue"[All Fields]) OR "methylene blue"[All Fields]) OR "Methylene Blue"[Mesh])
