# Supplementary material for: Efficacy and safety of methylene blue in the treatment of malaria: a systematic review
Source: BMC Med. 2018 Apr 25;16:59. doi: 10.1186/s12916-018-1045-3 (PMC5979000; doi:10.1186/s12916-018-1045-3)
Supplement: Supplementary file 2 — A list of all the records screened after duplicates were removed (N = 474). (PDF 329 kb) [file 12916_2018_1045_MOESM2_ESM.pdf]

# A list of total records screened after duplicates were removed (N=474)

## Part I. Records from all electronic databases after duplicates were removed (n=359)


1. (2000). "JAMA 100 years ago: methylene blue in grave malaria cachexia." Jama **283**(21): 2765.
2. Abdul-Ghani, R. and J. C. Beier (2014). "Strategic use of antimalarial drugs that block falciparum malaria parasite transmission to mosquitoes to achieve local malaria elimination." Parasitology Research **113**(10): 3535-3546.
3. Abelin, I. (1955). "Activation of thyroid hormone by methylene blue." Biochem Zeitschr **326**(3): 164-171.
4. Ademowo, O. G., C. M. Nneji and A. D. Adedapo (2007). "In vitro antimalarial activity of methylene blue against field isolates of Plasmodium falciparum from children in Southwest Nigeria." Indian J Med Res **126**(1): 45-49.
5. Adjalley, S. H., G. L. Johnston, T. Li, R. T. Eastman, E. H. Ekland, A. G. Eappen, A. Richman, B. K. L. Sim, M. C. S. Lee, S. L. Hoffman and D. A. Fidock (2011). "Quantitative assessment of Plasmodium falciparum sexual development reveals potent transmissionblocking activity by methylene blue." Proceedings of the National Academy of Sciences of the United States of America **108**(47): E1214-E1223.
6. Adler, E. and H. von Euler (1935). "tiber die Komponenten der Dehydrasesysteme." Hoppe Seyler S Zeitschr Physiol Chem **232**(1/2): 6-9.
7. Ager, A. L., M. Aviles, R. Boodoo, J. W. Anderson, G. D. Heffernan, P. E. Krasucki, K. W. Saionz, G. A. Schiehser, H. M. Shieh, W. Zhao, G. W. Birrell, M. Chavchich, M. D. Edstein, G. D. Shanks, L. R. Jacobus and D. P. Jacobus (2015). "In vivo efficacy of JPC-3210 and partner drugs for malaria eradication using the rodent-plasmodium berghei modified thompson test with an extended follow-up period." American Journal of Tropical Medicine and Hygiene **93 (4 Supplement)**: 78.
8. Akingbola, T. S., C. A. Ezekekwu, J. Yaria, S. L. Saraf, L. L. Hsu, R. S. Cooper, V. R. Gordeuk and B. O. Tayo (2015). "Assessment of bone marrow function in sickle cell anaemia patients using corrected reticulocyte counts." Blood **126 (23)**: 4581.
9. Akoachere, M., K. Buchholz, E. Fischer, J. Burhenne, W. E. Haefeli, R. H. Schirmer and K. Becker (2005). "In vitro assessment of methylene blue on chloroquine-sensitive and -resistant Plasmodium falciparum strains reveals synergistic action with artemisinins." Antimicrob Agents Chemother **49**(11): 4592-4597.
10. Ali, O. and C. Lindley (2012). "Case of methylene blue in severe obstetric sepsis." BJOG: An International Journal of Obstetrics and Gynaecology **119**: 110.
11. Allen, S. M., E. E. Lim, E. Jortzik, J. Preuss, H. H. Chua, J. I. MacRae, S. Rahlfs, K. Haeussler, M. T. Downton, M. J. McConville, K. Becker and S. A. Ralph (2015). "Plasmodium falciparum glucose-6-phosphate dehydrogenase 6-phosphogluconolactonase is a potential drug target." Febs Journal **282**(19): 3808-3823.
12. Aly, F. W. (1957). "Treatment of a case of severe NaCn poisoning with methylene blue and catalysin. [German]." Arztliche Wochenschrift **12**(44-45): 1014-1018.
13. Angus, B. (2014). "Novel anti-malarial combinations and their toxicity." Expert Review of Clinical Pharmacology **7**(3): 299-316.
14. Anonymous (2004). "The risks and benefits of treatments must be considered when treating or preventing malaria." Drugs and Therapy Perspectives **20**(12): 22-25.
15. Antunes, O. A. C. and A. C. Pinto (1984). "Malaria and antimalarial agents. [Portuguese]." Revista Brasileira de Farmacia **65**(1-3): 2-21.
16. Appel, W. and E. Werle (1956). "Photochemical inactivation of kallikrein by methylene blue. [German]." Zeitschrift fur Vitamin-, Hormon- und Fermentforschung **8**(1): 24-28.
17. Arias, D. G., E. L. Regner, A. A. Iglesias and S. A. Guerrero (2012). "Entamoeba histolytica thioredoxin reductase: Molecular and functional characterization, of its atypical properties." Biochimica Et Biophysica Acta-General Subjects **1820**(12): 1859-1866.
18. Atamna, H., M. Krugliak, G. Shalmiev, E. Deharo, G. Pescarmona and H. Ginsburg (1996). "Mode of antimalarial effect of methylene blue and some of its analogues on Plasmodium falciparum in culture and their inhibition of P. vinckei petteri and P. yoelii nigeriensis in vivo." Biochem Pharmacol **51**(5): 693-700.
19. Atamna, H., G. Pascarmona and H. Ginsburg (1994). "Hexose-monophosphate shunt activity in intact Plasmodium falciparum-infected erythrocytes and in free parasites." Molecular & Biochemical Parasitology **67**(1): 79-89.

20. Badesou, A. (1948). "Not Available. [Polyglot]." Wiener medizinische Wochenschrift (1946) **98**(33-34): 370.
21. Bahamontes-Rosa, N., M. G. Gomez-Lorenzo, J. Lelievre, A. Rodriguez Alejandre, M. J. Almela, S. Lozano, E. Herreros and F. J. Gamo (2016). "A novel validated assay to support the discovery of new anti-malarial gametocytocidal agents." Malaria Journal **15 (1) (no pagination)**(385).
22. Baird, K. J., J. D. Maguire and R. N. Price (2012). Diagnosis and Treatment of Plasmodium vivax Malaria. 24-28 Oval Road, London NW1 7DX, United Kingdom, Academic Press**:** 203-270.
23. Banasehak, H. and A. K. Hanel (1964). "Mechanism of fixation of methylene-blue to the surface of erythrocytes. [German]." Acta Biologica et Medica Germanica **12**(1): 90-103.
24. Barcia, J. J. (2007). "The Giemsa stain: its history and applications." Int J Surg Pathol **15**(3): 292-296.
25. Barnes, M. G. and H. Polet (1969). "The influence of methylene blue on the pentose phosphate pathway in erythrocytes of monkeys infected with Plasmodium knowlesi." The Journal of laboratory and clinical medicine **74**(1): 1-11.
26. Bartel, H. (1947). "The diagnosis of rabies. [German]." Zentralblatt fur Bakteriologie, Parasitenkunde, Infektionskrankheiten und Hygiene **2. Abteilung. 152**(3-4): 155-159.
27. Bauer, H. (1952). "Staining of CSF cells with a mixture of methylene blue and propylene glycol. [German]." Klinische Wochenschrift **30**(25-26): 612.
28. Becker, K., S. Rahlfs, C. Nickel and R. H. Schirmer (2003). "Glutathione: Functions and metabolism in the malarial parasite Plasmodium falciparum." Biological Chemistry **384**(4): 551-566.
29. Becker, W. A. (1933). "Vitalbeobachtungen iiber den Einfluss von Methylenblau und Neutralrot auf den Verlauf von Karyo- und Zytokinese. Beitrag zur Pathologie der Mitose." Cytologia **4**(2): 135-157.
30. Begon, E., O. Chosidow and P. Wolkenstein (2004). "Dipsulone. [French]." Annales de Dermatologie et de Venereologie **131**(12): 1062-1073.
31. Belorgey, D., D. A. Lanfranchi and E. Davioud-Charvet (2013). "1,4-Naphthoquinones and Other NADPH-Dependent Glutathione Reductase-Catalyzed Redox Cyclers as Antimalarial Agents." Current Pharmaceutical Design **19**(14): 2512-2528.
32. Bender, F. (1959). "The diagnostic evaluation of dye curves in congenital cardiac disease with left-right shunt using methylene blue. [German]." Zeitschrift fur Kreislaufforschung: 402-412.
33. Berg, H., F. A. Gollmick, H. E. Jacob and H. Triebel (1972). "Sensitized photooxidation through methylene blue, thiopyronine, and pyronine. II. Physicochemical bases for the photodynamic effectiveness of thiopyronine. [German]." Photochemistry and photobiology **16**(2): 125-138.
34. Betke, K. (1962). "The congenital red cell- and haemoglobin anomalies. [German]." Monatsschrift fur Kinderheilkunde **110**(3): 159-163.
35. Beutler, E. (1996). "G6PD: Population genetics and clinical manifestations." Blood Reviews **10**(1): 45-52.
36. Bezrukov, A. V. (2017). "Romanowsky staining, the Romanowsky effect and thoughts on the question of scientific priority." Biotech Histochem: 1-7.
37. Bielitza, M., D. Belorgey, K. Ehrhardt, L. Johann, D. A. Lanfranchi, V. Gallo, E. Schwarzer, F. Mohring, E. Jortzik, D. L. Williams, K. Becker, P. Arese, M. Elhabiri and E. Davioud-Charvet (2015). "Antimalarial NADPH-Consuming Redox-Cyclers As Superior Glucose-6-Phosphate Dehydrogenase Deficiency Copycats." Antioxidants & Redox Signaling **22**(15): 1337-1351.
38. Biot, C., H. Bauer, R. H. Schirmer and E. Davioud-Charvet (2004). "5-Substituted tetrazoles as bioisosteres of carboxylic acids. Bioisosterism and mechanistic studies on glutathione reductase inhibitors as antimalarials." Journal of Medicinal Chemistry **47**(24): 5972-5983.
39. Biot, C. and K. Chibale (2006). "Novel approaches to antimalarial drug discovery." Infectious Disorders - Drug Targets **6**(2): 173-204.
40. Birkholtz, L. M., T. L. Coetzer, D. Mancama, D. Leroy and P. Alano (2016). "Discovering New Transmission-Blocking Antimalarial Compounds: Challenges and Opportunities." Trends in Parasitology **32**(9): 669-681.
41. Birkholtz, L. M., D. Le Roy, J. Reader, M. Botha, D. Mancama and T. L. Coetzer (2014). "Differential activity of novel gametocytocidal compounds: Drug mode-of-action and ex vivo efficacy." Malaria Journal **13**: S14.
42. Bock, M. (1947). "Formation of Heinz bodies and methaemoglobin by methylene blue. [German]." Naunyn-Schmiedebergs Archiv fur experimentelle Pathologie und Pharmakologie **204**(6): 595-603.
43. Boroff, D. A. and R. W. Gronemyer (1945). "Technique for staining malaria smears." Bull U S Army Med Dept **88**: 113-114.
44. Bountogo, M., A. Zoungrana, B. Coulibaly, C. Klose, U. Mansmann, F. P. Mockenhaupt, J. Burhenne, G. Mikus, I. Walter-Sack, R. Heiner Schirmer, A. Sie, P. Meissner and O. Muller (2010). "Efficacy of methylene blue monotherapy in semi-immune adults with uncomplicated falciparum malaria: A controlled trial in Burkina Faso." Tropical Medicine and International Health **15**(6): 713-717.
45. Bousquet, A. (1930). "La bilharziose dans le Nefzaoua. Etude sommaire." Arch Inst Pasteur Tunis **19**(4): 438-450.

46. Brendel, M. and R. W. Kaplan (1967). "Photodynamic mutation induction and inactivation in Serratia phages kappa by methylene blue and light. [German]." Molecular & general genetics : MGG **99**(2): 181-190.
47. Brewer, G. J., A. R. Tarlov and A. S. Alving (1960). "Methaemoglobin reduction test. A new, simple in vitro test for identifying primaquine-sensitivity." Bulletin of the World Health Organization **22**(6): 633-640.
48. Buchholz, K., S. Rahlfs, R. Heiner Schirmer, K. Becker and K. Matuschewski (2008). "Depletion of Plasmodium berghei plasmoredoxin reveals a non-essential role for life cycle progression of the malaria parasite." PLoS ONE **3 (6) (no pagination)**(e2474).
49. Buchholz, K., R. H. Schirmer, J. K. Eubel, M. B. Akoachere, T. Dandekar, K. Becker and S. Gromer (2008). "Interactions of methylene blue with human disulfide reductases and their orthologues from Plasmodium falciparum." Antimicrob Agents Chemother **52**(1): 183-191.
50. Buchwalow, I., W. Boecker and M. Tiemann (2014). "The contribution of Paul Ehrlich to histochemistry: A tribute on the occasion of the centenary of his death." Virchows Archiv **466**(1): 111-116.
51. Burgemeister, G. (1952). "Synchronous determination of the circulation time with the aid of ether-methylene blue in cases of congenital cardiac malformation. [German]." Monatsschrift fur Kinderheilkunde **100**(4): 136-138.
52. Burkard, L., A. Scheuermann, J. Simithy and A. I. Calderon (2016). "Development of a functional assay to detect inhibitors of Plasmodium falciparum glutathione reductase utilizing liquid chromatography-mass spectrometry." Biomed Chromatogr **30**(4): 543-547.
53. Butterworth, A. S., T. S. Skinner-Adams, D. L. Gardiner and K. R. Trenholme (2013). "Plasmodium falciparum gametocytes: with a view to a kill." Parasitology **140**(14): 1718-1734.
54. Cabrera, M. and L. W. Cui (2015). "In Vitro Activities of Primaquine-Schizonticide Combinations on Asexual Blood Stages and Gametocytes of Plasmodium falciparum." Antimicrobial Agents and Chemotherapy **59**(12): 7650-7656.
55. Carson, P. E., R. Hohl, M. V. Nora, G. W. Parkhurst, T. Ahmad, S. Scanlan and H. Frischer (1981). "Toxicology of the 8-aminoquinolines and genetic factors associated with their toxicity in man." Bulletin of the World Health Organization **59**(3): 427-437.
56. Chandra, R. and S. K. Puri (2015). "Arteether resistance reversal by ketoconazole/fluconazole in rodent malaria parasite Plasmodium vinckei." Parasitology Research **114**(3): 1239-1243.
57. Cobbold, S. A., H. H. Chua, B. Nijagal, D. J. Creek, S. A. Ralph and M. J. McConville (2016). "Metabolic Dysregulation Induced in Plasmodium falciparum by Dihydroartemisinin and Other Front-Line Antimalarial Drugs." J Infect Dis **213**(2): 276-286.
58. Coleman, M. D. (2007). "Improvement of patient tolerance to dapsone: Current and future developments." Dermatology Online Journal **13 (4) (no pagination)**(18).
59. Coulibaly, B., M. Pritsch, M. Bountogo, P. Meissner, E. Nebié, C. Klose, M. Kieser, N. Berens-Riha, A. Wieser, S. Sirima, J. Breitkreutz, R. Schirmer, A. Sié, F. Mockenhaupt, C. Drakeley, T. Bousema and O. Müller (2015) "Efficacy and safety of triple combination therapy with artesunate-amodiaquine-methylene blue for falciparum malaria in children: a randomized controlled trial in Burkina Faso." The Journal of infectious diseases **211**, 689-697 DOI: 10.1093/infdis/jiu540.
60. Coulibaly, B., A. Zoungrana, F. Mockenhaupt, R. Schirmer, C. Klose, U. Mansmann, P. Meissner and O. Müller (2009) "Strong gametocytocidal effect of methylene blue-based combination therapy against falciparum malaria: a randomised controlled trial." PloS one **4**, e5318 DOI: 10.1371/journal.pone.0005318.
61. Cremer-Bartels, G. (1964). "Effect of methylene blue, daylight and cortisone on lenses of cattle in vitro. [German]." Albrecht V **Graefes Arch. Ophthal. 167**(6): 537-540.
62. Curd, F. H. S. (1947). "Researches on the chemotherapy of malaria II. The chemical approach." Glasgow Medical Journal **28**(3): 73-79.
63. D'Alessandro, S., N. Basilico, Y. Corbett, S. Parapini, F. Silvestrini, K. Dechering, T. Bianchi, P. Verducci, R. Sauerwein, P. Alano and D. Taramelli (2012). "A new P. Falciparum gametocyte drug screening assay based on pLDH detection." Malaria Journal **11**: S11.
64. D'Alessandro, S., F. Silvestrini, K. Dechering, Y. Corbett, S. Parapini, M. Timmerman, L. Galastri, N. Basilico, R. Sauerwein, P. Alano and D. Taramelli (2013). "A Plasmodium falciparum screening assay for anti-gametocyte drugs based on parasite lactate dehydrogenase detection." J Antimicrob Chemother **68**(9): 2048-2058.
65. De Asis Alcantara Nicolas, F., R. P. Mesonero, V. M. Molero, M. A. P. Nieto, C. S. Herreros, A. B. Ruiz, A. M. Fuentes, E. M. Alcalde, C. P. Monroy and E. De Eusebio Murillo (2016). "Irritant contact dermatitis from "miracle mineral solution"." Journal of the American Academy of Dermatology **1)**: AB92.
66. Dechy-Cabaret, O. and F. Benoit-Vical (2012). "Effects of Antimalarial Molecules on the Gametocyte Stage of Plasmodium falciparum: The Debate." Journal of Medicinal Chemistry **55**(23): 10328-10344.

67. Deharo, E., R. N. Garcia, P. Oporto, A. Gimenez, M. Sauvain, V. Jullian and H. Ginsburg (2002). "A non-radiolabelled ferriprotoporphyrin IX biomineralisation inhibition test for the high throughput screening of antimalarial compounds." Exp Parasitol **100**(4): 252-256.
68. Del Frari, B., H. Piza-Katzer, T. Schoeller and G. Wechselberger (2007). "External lymph fistula of the lower leg: Intraoperative detection and therapy by interdigifal injection of methylen blue. [German]." Phlebologie **36**(5): 267-271.
69. Delves, M. J., A. Ruecker, U. Straschil, J. Lelievre, S. Marques, M. J. Lopez-Barragan, E. Herreros and R. E. Sinden (2013). "Male and female Plasmodium falciparum mature gametocytes show different responses to antimalarial drugs." Antimicrob Agents Chemother **57**(7): 3268-3274.
70. Demandt, M. and H. Wandt (1996). "Successful treatment of ifosfamide-associated encephalopathy with methylene blue. [German]." Deutsche Medizinische Wochenschrift **121**(17): 575.
71. Deslauriers, R., K. Butler and I. C. P. Smith (1987). "Oxidant stress in malaria as probed by stable nitroxide radicals in erythrocytes infected with Plasmodium berghei. The effects of primaquine and chloroquine." Biochimica et Biophysica Acta - Molecular Cell Research **931**(3): 267-275.
72. Dhaliwal, G., P. A. Cornett and L. M. Tierney Jr (2004). "Hemolytic anemia." American Family Physician **69**(11): 2599-2606.
73. Donaldson, P. T., A. Russo, C. Reynolds and R. D. Lillie (1978). "Borax Methylene Blue a Spectroscopic and Staining Study." Stain Technology **53**(4): 225-228.
74. Dormoi, J., S. Briolant, C. Desgrouas and B. Pradines (2013). "Efficacy of proveblue (methylene blue) in an experimental cerebral malaria murine model." Antimicrob Agents Chemother **57**(7): 3412-3414.
75. Dormoi, J., A. Pascual, S. Briolant, R. Amalvict, S. Charras, E. Baret and B. Pradines (2012). "Proveblue (methylene blue) as an antimalarial agent." American Journal of Tropical Medicine and Hygiene **1)**: 45.
76. Dormoi, J. and B. Pradines (2013). "Dose responses of proveblue methylene blue in an experimental murine cerebral malaria model." Antimicrob Agents Chemother **57**(8): 4080-4081.
77. Dormoi, J., H. Savini, R. Amalvict, E. Baret and B. Pradines (2014). "In vitro interaction of lumefantrine and piperaquine by atorvastatin against Plasmodium falciparum." Malaria Journal **13**: 6.
78. Dutta, G., S. Nagarajan, L. J. Lapidus and P. B. Lillehoj (2016). "Enzyme-free electrochemical immunosensor based on methylene blue and the electro-oxidation of hydrazine on Pt nanoparticles." Biosens Bioelectron.
79. Dwelle, T. L. (1995). "Inadequate basic preventive health measures: Survey of missionary children in sub-Saharan Africa." Pediatrics **95**(5): 733-737.
80. Ehrhardt, K., E. Davioud-Charvet, H. Ke, A. B. Vaidya, M. Lanzer and M. Deponte (2013). "The antimalarial activities of methylene blue and the 1,4-naphthoquinone 3-[4-(trifluoromethyl)benzyl]-menadione are not due to inhibition of the mitochondrial electron transport chain." Antimicrob Agents Chemother **57**(5): 2114-2120.
81. Ehringer, H., O. Hornykiewicz and K. Lechner (1961). "The action of methylene blue on monoamine oxidase and the catecholamines and the metabolism of 5-hydroxytryptamine in brain [English summ.]." Arch Exptl Pathol U Pharmakol **241**(1/5): 568-582.
82. Eisenstein, M. (2012). "Drug development: Holding out for reinforcements." Nature **484**(7395 SUPPL.): S16-S18.
83. Eliaszewicz-Branicka, M. (1948). "A contribution to the physiopathology of leukergy." Ann Univ Mariae Curie Sklodowska Sect D Med **3**(3): 215-236.
84. Euler, H. and A. Olander (1928). "Catalytic hastening of the oxido-reduction formic acid-methylene blue." Zeitschr Physikal Chem Abt A **137**(1/4): 29-44.
85. Evora, P. R. B. (2013). "Methylene blue for the treatment of refractory anaphylaxis without hypotension." American Journal of Emergency Medicine **31**(4): 753.
86. Falla Velasquez, L. A. (1949). "Comparative study between '4888' and 'SN 7618' in the treatment of malaria. [Italian]." Med **Cir. Bogota 13**(6): 183-205.
87. Farber, P. M., L. D. Arscott, C. H. Williams, Jr., K. Becker and R. H. Schirmer (1998). "Recombinant Plasmodium falciparum glutathione reductase is inhibited by the antimalarial dye methylene blue." FEBS Lett **422**(3): 311-314.
88. Faulhaber, H. D. and H. Spinner (1965). "The influence of liver perfusion on the reduction of methemoglobin. I. Studies on erythrocytes of different species and the influence of methylene blue [Engl. and Russ. summ. ]." Acta Biol Med Ger **14**(3/4): 310-317.
89. Fenton, J. C. B. J. (1945). "Innes A staining method for malaria parasites in thick blood-films." Trans Roy Soc Trop Med And Hyg **39**(1): 87-90.
90. Fernandez-Alvaro, E., W. D. Hong, G. L. Nixon, P. M. O'Neill and F. Calderon (2016). "Antimalarial Chemotherapy: Natural Product Inspired Development of Preclinical and Clinical Candidates with Diverse Mechanisms of Action." Journal of Medicinal Chemistry **59**(12): 5587-5603.
91. Ferrant, M. (1946). "Methaemoglobinaemia Two cases in newborn infants caused by nitrates in well water." Journal of pediatrics **29**(5): 585-592.

92. Fidock, D. A. (2011). "Quantitative assessment of Plasmodium falciparum sexual development reveals potent transmission-blocking activity of the synthetic dye methylene blue." American Journal of Tropical Medicine and Hygiene **1)**: 216.
93. Field, J. W. (1941). "Further note on a method of staining malarial parasites in thick blood films." Trans Roy Soc Trop Med And Hyg **35**(1): 35-42.
94. Flannery, E. L., D. A. Fidock and E. A. Winzeler (2013). "Using genetic methods to define the targets of compounds with antimalarial activity." Journal of Medicinal Chemistry **56**(20): 7761-7771.
95. Fleischer, B. (2004). "Editorial: 100 Years ago - Giemsa's solution for staining of plasmodia." Tropical Medicine and International Health **9**(7): 755-756.
96. Floch, H. (1945). "Economical treatment of malaria with "activated quinine."." Bull Soc Path Exot **38**(11/12): 327-341.
97. Foedinger, A., T. J. Luger, G. Erdoes, L. Luzzatto and C. J. F. Van Noorden (2016). "Anaesthesia recommendations for patients suffering from Glucose-6-phosphate dehydrogenase deficiency." Anasthesiologie und Intensivmedizin **57**(4): S123-S135.
98. Francis, S. E., D. J. Sullivan Jr and D. E. Goldberg (1997). Hemoglobin metabolism in the malaria parasite Plasmodium falciparium. 4139 El Camino Way, P.O. Box 10139, Palo Alto CA 94306, United States, Annual Reviews Inc.**:** 97-123.
99. Frank, H., G. Werner, E. Auermann and R. Meyer (1985). "Sources of error and optimization of the methylene blue procedure. CdS suspensions as reference samples for the ring test. [German]." Zeitschrift fur die gesamte Hygiene und ihre Grenzgebiete **31**(10): 592-594.
100. Frankenburg, F. R. and R. J. Baldessarini (2008). "Neurosyphilis, malaria, and the discovery of antipsychotic agents." Harv Rev Psychiatry **16**(5): 299-307.
101. Froes, H. P. (1934). "Methylene blue in the treatment of malaria." Riv Malariol **13**(4): 484-486.
102. Fulton, J. D. and A. Voller (1964). "EVALUATION OF IMMUNOFLUORESCENT AND DIRECT AGGLUTINATION METHODS FOR DETECTION OF SPECIFIC TOXOPLASMA ANTIBODIES." Br Med J **2**(5418): 1173-1175.
103. Gallo, V., E. Schwarzer, S. Rahlfs, R. H. Schirmer, R. van Zwieten, D. Roos, P. Arese and K. Becker (2009). "Inherited glutathione reductase deficiency and Plasmodium falciparum malaria - A case study." PLoS ONE **4 (10) (no pagination)**(e7303).
104. Garavito, G., S. Bertani, M. Quiliano, A. Valentin, I. Aldana and E. Deharo (2012). "The in vivo antimalarial activity of methylene blue combined with pyrimethamine, chloroquine and quinine." Mem Inst Oswaldo Cruz **107**(6): 820-823.
105. Garavito, G., S. Bertani, J. Rincon, S. Maurel, M. C. Monje, I. Landau, A. Valentin and E. Deharo (2007). "Blood schizontocidal activity of methylene blue in combination with antimalarials against plasmochum falciparum." Parasite **14**(2): 135-140.
106. Gebru, T., B. Mordmuller and J. Held (2014). "Effect of Fluorescent Dyes on In Vitro-Differentiated, Late-Stage Plasmodium falciparum Gametocytes." Antimicrobial Agents and Chemotherapy **58**(12): 7398-7404.
107. Gensini, G. F., A. A. Conti and D. Lippi (2007). "The contributions of Paul Ehrlich to infectious disease." J Infect **54**(3): 221-224.
108. Gensthaler, B. M. (2004). "Ehrlich's methylene blue: Blue dye against malaria. [German]." Pharmazeutische Zeitung **149**(39): 22-23.
109. Giemsa, G., W. Weise and C. Tropp (1926). "No English Title Available." Arch Schiffs U Tropen Hyg **30**(8): 334-347.
110. Giselsson, W. (1952). "Staining of malaria parasites with Manson's solution. [Dutch]." Nordisk Medicin **47**(11): 367.
111. Glogner, P., H. P. Wolf and H. Holzer (1960). "The influence of methylene blue on the glycolysis and respiration of ascites-tumour cells. [German]." Biochemische Zeitschrift **332**(5): 407-415.
112. Gollmick, F. A. and H. Berg (1968). "Sensitized photo-oxidation by methylene blue, thiopyronine and pyronine. I. Flash-photo-oxidation of p-diaminotoluene. [German]." Photochemistry and photobiology **7**(5): 471-475.
113. Gollmick, F. A. and H. Berg (1972). "Methylene Blue thyropyronin and pyronin-sensitized photooxidation. 3. Mechanism of photosensitized oxidation of guanosines by thiopyronin. [German]." Photochemistry and photobiology **16**(5): 447-453.
114. Gollmick, F. A. and H. Lang (1981). "Study of the photodynamic effect of methylene blue in chromatin. [German]." Dermatologische Monatsschrift **167**(10): 627.
115. Gottsegen, G., I. Szam and M. Csornay (1958). "Effect of methylene blue on experimental acute pulmonary edema. [German]." Naunyn-Schmiedebergs Archiv fur experimentelle Pathologie und Pharmakologie **234**(2): 126-132.
116. Grabenko, I. K. (1946). "[Combined treatment of malaria with methylene blue and glucose]." Vrach Delo(5): 213-218.

117. Grellier, P., J. Sarlauskas, Z. Anusevicius, A. Maroziene, C. Houee-Levin, J. Schrevel and N. Cenas (2001). "Antiplasmodial activity of nitroaromatic and quinoidal compounds: Redox potential vs inhibition of erythrocyte glutathione reductase." Archives of Biochemistry & Biophysics **393**(2): 199-206.
118. Gureev, A. P., M. Y. Syromyatnikov, T. M. Gorbacheva, A. A. Starkov and V. N. Popov (2016). "Methylene blue improves sensorimotor phenotype and decreases anxiety in parallel with activating brain mitochondria biogenesis in mid-age mice." Neuroscience Research **113**: 19-27.
119. Gurr, E. (1964). "ROLE OF EOSIN IN ROMANOWSKY STAINING OF MALARIA NUCLEUS." Nature **202**: 1022-1023.
120. Gut, F., W. Schiek, W. E. Haefeli, I. Walter-Sack and J. Burhenne (2008). "Cation exchange resins as pharmaceutical carriers for methylene blue: binding and release." Eur J Pharm Biopharm **69**(2): 582-587.
121. Haenel, A. (1964). "ON THE TOXIC EFFECTS OF METHYLENE BLUE ON ERYTHROCYTES IN VIVO. [German]." Acta biologica et medica Germanica **12**: 644-654.
122. Hamblin, M. R. and T. Hasan (2004). "Photodynamic therapy: A new antimicrobial approach to infectious disease?" Photochemical and Photobiological Sciences **3**(5): 436-450.
123. Hanel, A. (1964). "Toxic effects of methylene blue on erythrocytes in vivo. [German]." Acta Blol **Med.Germ. 12**(6): 644-654.
124. Hanf, U., S. Heinrich and Legler (1953). "Examination of the sensitivity of the stimulator of actinomycosis to antibiotics (penicillin, streptomycin Aureomycin, chloromycetin, Terramycin) and methylene blue." Arch Hyg A Bakt **137**(7): 527-538.
125. Hanscheid, T., T. J. Egan and M. P. Grobusch (2007). "Haemozoin: from melatonin pigment to drug target, diagnostic tool, and immune modulator." Lancet Infectious Diseases **7**(10): 675-685.
126. Hansen, F. K., T. S. Skinner-Adams, S. Duffy, L. Marek, S. D. M. Sumanadasa, K. Kuna, J. Held, V. M. Avery, K. T. Andrews and T. Kurz (2014). "Synthesis, antimalarial properties, and sar studies of alkoxyurea-based HDAC inhibitors." ChemMedChem **9**(3): 665-670.
127. Hardel, M. and F. Todt (1964). "The influence of methylene blue and brilliant cresyl blue on the growth of the Ehrlich ascites tumor." Naturwissenschaften **51**(16): 392-393.
128. Harris, F., L. K. Chatfield and D. A. Phoenix (2005). "Phenothiazinium based photosensitisers - Photodynamic agents with a multiplicity of cellular targets and clinical applications." Current Drug Targets **6**(5): 615-627.
129. Harwaldt, P., S. Rahlfs and K. Becker (2002). "Glutathione S-transferase of the malarial parasite Plasmodium falciparum: Characterization of a potential drug target." Biological Chemistry **383**(5): 821-830.
130. Hasart, E., G. Jacobasch and S. Rapoport (1972). "Behavior of the NAD (P) level in human erythrocytes with variation of the pH value and under the influence of methylene blue. [German]." Acta Biologica et Medica Germanica **28**(4): 603-613.
131. Haynes, R. K., W. C. Chan, H. N. Wong, K. Y. Li, W. K. Wu, K. M. Fan, H. H. Sung, I. D. Williams, D. Prosperi, S. Melato, P. Coghi and D. Monti (2010). "Facile oxidation of leucomethylene blue and dihydroflavins by artemisinins: relationship with flavoenzyme function and antimalarial mechanism of action." ChemMedChem **5**(8): 1282-1299.
132. Haynes, R. K., K. W. Cheu, H. W. Chan, H. N. Wong, K. Y. Li, M. M. Tang, M. J. Chen, Z. F. Guo, Z. H. Guo, K. Sinniah, A. B. Witte, P. Coghi and D. Monti (2012). "Interactions between artemisinins and other antimalarial drugs in relation to the cofactor model--a unifying proposal for drug action." ChemMedChem **7**(12): 2204-2226.
133. Haynes, R. K., K. W. Cheu, K. Y. Li, M. M. Tang, H. N. Wong, M. J. Chen, Z. F. Guo, Z. H. Guo, P. Coghi and D. Monti (2011). "A partial convergence in action of methylene blue and artemisinins: antagonism with chloroquine, a reversal with verapamil, and an insight into the antimalarial activity of chloroquine." ChemMedChem **6**(9): 1603-1615.
134. Haynes, R. K., K. W. Cheu, D. N'Da, P. Coghi and D. Monti (2013). "Considerations on the mechanism of action of artemisinin antimalarials: Part 1 - The 'carbon radical' and 'heme' hypotheses." Infectious Disorders - Drug Targets **13**(4): 217-277.
135. Haynes, R. K., K. W. Cheu, M. M. Tang, M. J. Chen, Z. F. Guo, Z. H. Guo, P. Coghi and D. Monti (2011). "Reactions of antimalarial peroxides with each of leucomethylene blue and dihydroflavins: flavin reductase and the cofactor model exemplified." ChemMedChem **6**(2): 279-291.
136. Held, J., S. Jeyaraj and A. Kreidenweiss (2015). "Antimalarial compounds in Phase II clinical development." Expert Opin Investig Drugs **24**(3): 363-382.
137. Helmcke, R. (1956). "The sensitization of Escherichia coli to irradiation with shortwave ultraviolet light, wave-length 2537 A, by methylene blue." Naturwiss **43**(5): 111-112.
138. Hemingway, J., R. Shretta, T. N. C. Wells, D. Bell, A. A. Djimde, N. Achee and G. Qi (2016). "Tools and Strategies for Malaria Control and Elimination: What Do We Need to Achieve a Grand Convergence in Malaria?" PLoS Biology **14 (3) (no pagination)**(e1002380).

139. Herzberg, K., K. Reuss and R. Dahn (1963). "Photodynamic action and virus inactivation through methylene blue and thiopyronin." Naturwissenschaften **50**(10): 376-377.
140. Horobin, R. W. (2011). "How Romanowsky stains work and why they remain valuable - Including a proposed universal Romanowsky staining mechanism and a rational troubleshooting scheme." Biotechnic and Histochemistry **86**(1): 36-51.
141. Houwen, B. (2002). "Blood film preparation and staining procedures." Clin Lab Med **22**(1): 1-14, v.
142. Howarth, J. and D. G. Lloyd (2001). "Redox systems as conduits for antimalarial compounds." Journal of Antimicrobial Chemotherapy **47**(1): 122-124.
143. Howland, R. H. (2016). "Methylene Blue The Long and Winding Road From Stain to Brain: Part 2." Journal of Psychosocial Nursing and Mental Health Services **54**(10): 21-26.
144. Hubl, P. and W. Zischka (1960). "ADDITION of METHYLENE BLUE to THE GASTRIC TEST MEAL -. [German]." Arztl.Lab **6**(1): 10-14.
145. Hue, N. T., J. P. Charlieu, T. T. H. Chau, N. Day, J. J. Farrar, T. T. Hien and S. J. Dunstan (2009). "Glucose-6-phosphate dehydrogenase (G6PD) mutations and haemoglobinuria syndrome in the vietnamese population." Malaria Journal **8 (1) (no pagination)**(152).
146. Huhnerfeld, J. (1947). "On the influence of porphyrins on the resorption and the decolorization of methylene blue blisters. [German]." Klinische Wochenschrift **24-25**(27-28): 264.
147. Hulser, D. F. (1968). "Localization of reductive sites in bacteria by methylenblue-silver (Argochrome) with the electron microscope. [German]." Biophysik **5**(3): 165-182.
148. Ignatushchenko, M. V., R. W. Winter, H. P. Bachinger, D. J. Hinrichs and M. K. Riscoe (1997). "Xanthones as antimalarial agents; studies of a possible mode of action." FEBS Letters **409**(1-2): 67-73.
149. Imperatore, C., M. Persico, A. Aiello, P. Luciano, M. Guiso, M. F. Sanasi, D. Taramelli, S. Parapini, G. Cebrian-Torrejon, A. Domenech-Carbo, C. Fattorusso and M. Menna (2015). "Marine inspired antiplasmodial thiazinoquinones: synthesis, computational studies and electrochemical assays." Rsc Advances **5**(86): 70689-70702.
150. Ito, S. (1941). "Uber die hemmende Wirk-ung von Methylenblau bei experimenteller Hepatomentsteh-ung. II." Gann **35**(3): 167-185.
151. Jaeger, A., P. Sauder, J. Kopferschmitt and F. Flesch (1987). "Clinical features and management of poisoning due to antimalarial drugs." Medical Toxicology and Adverse Drug Experience **2**(4): 242-273.
152. Jakuboviu, A. and J. Nesina (1963). "Effects of methylene blue administered by various routes on amine oxidase activity in rat liver and brain. [German]." Arzneimittel-Forschung **3**(2): 134-143.
153. Joanny, F., J. Held and B. Mordmuller (2012). "In Vitro activity of fluorescent dyes against asexual blood stages of Plasmodium falciparum." Antimicrobial Agents and Chemotherapy **56**(11): 5982-5985.
154. Johnston, G. L., P. W. Gething, S. I. Hay, D. L. Smith and D. A. Fidock (2014). "Modeling Within-Host Effects of Drugs on Plasmodium falciparum Transmission and Prospects for Malaria Elimination." Plos Computational Biology **10**(1): 16.
155. Kagan, I. G. and K. W. Walls (1981). Protozoa and Helminths. Milgrom, F., C. J. Abeyounis and K. Kano. Principles of Immunological Diagnosis in Medicine. Xvi+520p. Lea and Febiger: Philadelphia, Pa., USA. Illus. P275-285, 1981.
156. Kakai, R. M., J. Nasimiyu and W. Odero (2011). "Low reliability of home-based diagnosis of malaria in a rural community in western Kenya." J Infect Dev Ctries **5**(1): 54-58.
157. Kasozi, D. M., S. Gromer, H. Adler, K. Zocher, S. Rahlfs, S. Wittlin, K. Fritz-Wolf, R. H. Schirmer and K. Becker (2011). "The bacterial redox signaller pyocyanin as an antiplasmodial agent: comparisons with its thioanalog methylene blue." Redox Rep **16**(4): 154-165.
158. Kass, L. (1977). "A note on reticulocytes." Archives of Pathology and Laboratory Medicine **101**(10): 514-515.
159. Kaufmann, S. H. E. (2008). "Paul Ehrlich: Founder of chemotherapy." Nature Reviews Drug Discovery **7**(5): 373.
160. Kholewski, R. and G. Lindemann (1954). "Application of the methylene blue reduction test in maternal milk banks. [German]." Zbl **Gynak. 76**(47): 2090-2093.
161. Kikuth, W. and I. Schilling (1944). "Chemotherapeutic expts. on typhus (Rickettsia mooseri) with methylene blue." Zentralbl Bakt I Abt Orig **151**: 293-302.
162. Kikuth, W. and C. Tropp (1927). "Bird malaria." Hamburg Univ Abhandl Gebiet Auslandsk **26**: 236-245.
163. Kirchmair, H. (1950). "The Giemsa staining method (azuremethylene blue-eosin staining). [German]." Forsch **Forscher Tiroler Arzteschule (1948-1950) 2**: 107-131.
164. Kiszewski, A. E. (2011). "Blocking Plasmodium falciparum malaria transmission with drugs: The gametocytocidal and sporontocidal properties of current and prospective antimalarials." Pharmaceuticals **4**(1): 44-68.
165. Kleeberg, J. (1972). "Staining of uric acid crystals with methylene blue. [German]." Zeitschrift fur Urologie und Nephrologie **65**(8): 619-629.

166. Kleemann, D. (1990). "Experimental investigations of photodynamic treatment of malignant tumors of oral cavity, pharynx, and larynx with photosensitizer Methylene blue. [German]." Laryngo- Rhino-Otologie **69**(8): 437-439.
167. Knuesel, O. (1946). "Not Available. [Polyglot]." Ophthalmologica **Journal international d'ophtalmologie. International journal of ophthalmology. Zeitschrift fur Augenheilkunde. 111**(45): 295-297.
168. Kohne, E. and E. Kleihauer (1974). "Heinz body formation in red cells of newborn infants. II. Clinical observations on methylene blue induced hemolytic Heinz body anemia. [German]." Monatsschrift fur Kinderheilkunde **122**(2): 56-59.
169. Komuro, H. (1934). "Uber das Verfahren mit einer neuen Farbungsmethode fur Krebszellen nach Ko-muro und dessen Applikationswert. II. Ein Doppelfarb-ungsverfahren mit Kongorot und Methylenblau zur Unterscheidung der Krebszellcharaktere." Gann **28**(3): 376-387.
170. Konig, K. and H. Meyer (1993). "Photodynamic activity of methylene blue. [German]." Aktuelle Dermatologie **19**(7): 195-198.
171. Koschuth, A., P. E. Spath-Schwalbe and K. Possinger (1996). "Methylene blue in ifosfamide-induced encephalopathy. [German]." Deutsche medizinische Wochenschrift (1946) **121**(39): 1210.
172. Krafts, K., E. Hempelmann and B. J. Oleksyn (2011). "In search of the malarial parasite: biographical sketches of the blood stain contributors." Parasitol Res **109**(3): 521-529.
173. Krafts, K., E. Hempelmann and A. Skorska-Stania (2012). "From methylene blue to chloroquine: a brief review of the development of an antimalarial therapy." Parasitol Res **111**(1): 1-6.
174. Krafts, K. P., E. Hempelmann and B. J. Oleksyn (2011). "The color purple: From royalty to laboratory, with apologies to Malachowski." Biotechnic and Histochemistry **86**(1): 7-35.
175. Krahl, K. P. and K. H. Fromming (1982). "Enteric control film tablets from an aqueous solution of a copolymer of vinyl acetate and crotonic acid. In vivo drug release from enteric coated tablets with methylene blue. [German]." Pharmazeutische Industrie **44**(10): 1084-1087.
176. Kruger, J. (1978). "Height localization in surgery of spinal region using labelling by methylene blue. [German]." Deutsche medizinische Wochenschrift (1946) **103**(10): 432.
177. Kumar, S., M. Guha, V. Choubey, P. Maity and U. Bandyopadhyay (2007). "Antimalarial drugs inhibiting hemozoin (beta-hematin) formation: A mechanistic update." Life Sciences **80**(9): 813-828.
178. Kumar, S., R. Kumari and R. Pandey (2015). "New insight-guided approaches to detect, cure, prevent and eliminate malaria." Protoplasma **252**(3): 717-753.
179. Kupfer, A., J. R. Idle, M. Quere, A. Ogouassangni, A. Bokossa, A. Perra, W. Van Damme, H. Stanley, A. Sherratt and D. M. Turnbull (1999). "Methylene blue and fatal encephalopathy from ackee fruit poisoning (multiple letters) [4]." Lancet **353**(9164): 1622-1624.
180. la Terza, C. N. (1944). "Inedita modalidade termica num caso com 45 graus de temperatura, no decurso de uma psicose malarica." Anais Paulistas Med E Cir **48**(1): 5-27.
181. Lanfranchi, D. A., D. Belorgey, T. Muller, H. Vezin, M. Lanzer and E. Davioud-Charvet (2012). "Exploring the trifluoromenadione core as a template to design antimalarial redox-active agents interacting with glutathione reductase." Organic & Biomolecular Chemistry **10**(24): 4795-4806.
182. Lanfranchi, D. A., E. Cesar-Rodo, B. Bertrand, H. H. Huang, L. Day, L. Johann, M. Elhabiri, K. Becker, D. L. Williams and E. Davioud-Charvet (2012). "Synthesis and biological evaluation of 1,4-naphthoquinones and quinoline-5,8-diones as antimalarial and schistosomicidal agents." Organic & Biomolecular Chemistry **10**(31): 6375-6387.
183. Lee, W. C., B. Russell, Y. L. Lau, M. Y. Fong, C. Chu, K. Sriprawat, R. Suwanarusk, F. Nosten and L. Renia (2013). "Giemsa-Stained Wet Mount Based Method for Reticulocyte Quantification: A Viable Alternative in Resource Limited or Malaria Endemic Settings." PLoS ONE **8 (4) (no pagination)**(e60303).
184. Lehmann, J. (1929). "The methylene blue method for determining dehydrogenation. 1. pH measurement, with the quinhydrone electrode, of succinodehydrogenase solutions at 37[degree] C. in presence of methylene blue." Skand Arch Physiol **55**(6): 307-315.
185. Leman, A. (1965). "Electrometrically and colorimetrically measured redox-potentials of fermenting yeast suspensions during potential drift. Studies with indigo carmine and methylene blue." Arch Mikrobiol **50**(4): 357-387.
186. Lembke, A., V. Kaufmann and H. Lagoni (1951). "The reduction of methylene blue by bacilli. [German]." Naturwissenschaften **38**(24): 563.
187. Lillie, R. D. (1978). "Romanowsky Malachowski Stains the So-Called Romanowsky Stain Malachowskis Use of Alkali Poly Chromed Methylene Blue for Malaria Plasmodia." Stain Technology **53**(1): 23-28.
188. Lillie, R. D., P. T. Donaldson and P. Pizzolato (1978). "Preparation of Eosinates and Giemsa Stains of Low Azure B Content from Hot Acid Di Chromate Oxidized Commercial Medicinal Methylene Blue." Stain Technology **53**(6): 337-344.

189. Lillie, R. D. and M. A. Roe (1942). "Studies on polychrome methylene blue." Stain Technol **17**(2): 57-63.
190. Lim, S. and J. H. Prieto (2015). "Glutathione Reductase of Plasmodium Falciparum as an Antimalarial Drug Target of Methylene Blue." Biophysical Journal **108**(2): 55A-56A.
191. Lindholm, P. F., K. Annen and G. Ramsey (2011). "Approaches to minimize infection risk in blood banking and transfusion practice." Infectious Disorders - Drug Targets **11**(1): 45-56.
192. Loffler, E. and R. Rigler (1926). "Respiration of bacteria through methylene blue reduction." Centralbl Bakt **99**(1B): 1-16.
193. Lopez Antunano, F. J. (1959). "[Microscopy in human malaria." Salud Publ Mexico **1**(2): 139-168.
194. Lopez-Miranda, J. L., S. E. Borjas-Garcia, R. Esparza and G. Rosas (2016). "Synthesis and Catalytic Evaluation of Silver Nanoparticles Synthesized with Aloysia triphylla Leaf Extract." Journal of Cluster Science **27**(6): 1989-1999.
195. Lotharius, J., F. J. Gamo-Benito, I. Angulo-Barturen, J. Clark, M. Connelly, S. Ferrer-Bazaga, T. Parkinson, P. Viswanath, B. Bandodkar, N. Rautela, S. Bharath, S. Duffy, V. M. Avery, J. J. Mohrle, R. K. Guy and T. Wells (2014). "Repositioning: The fast track to new anti-malarial medicines?" Malaria Journal **13 (1) (no pagination)**(143).
196. Lucantoni, L. and V. Avery (2012). "Whole-cell in vitro screening for gametocytocidal compounds." Future Medicinal Chemistry **4**(18): 2337-2360.
197. Lucantoni, L., D. A. Fidock and V. M. Avery (2016). "Luciferase-based, high-throughput assay for screening and profiling transmission-blocking compounds against Plasmodium falciparum gametocytes." Antimicrobial Agents and Chemotherapy **60**(4): 2097-2107.
198. Luond, R. M., J. H. McKie, K. T. Douglas, M. J. Dascombe and J. Vale (1998). "Inhibitors of glutathione reductase as potential antimalarial drugs. Kinetic cooperativity and effect of dimethyl sulphoxide on inhibition kinetics." J Enzyme Inhib **13**(5): 327-345.
199. MacLennan, S. and J. A. J. Barbara (2006). "Risks and side effects of therapy with plasma and plasma fractions." Best Practice and Research: Clinical Haematology **19**(1): 169-189.
200. Maffioli, C., H. Louvet and H. Salas (1978). "Importance of vital staining with methylene blue in examining duodenal ulcers. [German]." Zeitschrift fur Gastroenterologie **16**(11): 696-697.
201. Makovitzky, J. (1978). "Investigation of the anisotropy of glycocalyx stained with 1.9-dimethyl methylene blue and N,N'-diethylpseudoisocyanine chloride. [German]." Histochemistry **59**(1): 55-66.
202. Mameghani, A. (2011). "[Intrathecal administration of methylene blue is obsolete]. [German]." Der Unfallchirurg **114**(6): 549.
203. Manganelli, G., U. Masullo, S. Passarelli and S. Filosa (2013). "Glucose-6-phosphate dehydrogenase deficiency: Disadvantages and possible benefits." Cardiovascular and Hematological Disorders - Drug Targets **13**(1): 73-82.
204. Manwell, R. D. and P. Feigelson (1948). "A modified method of preparing the J. S. B. stain." J **Lab. Clin. Med. St Louis 33**(6): 777-782.
205. Markl, B., T. Kerwel, H. Jahnig, M. Anthuber and H. Arnholdt (2008). "Lymph node preparation in colorectal cancer. Ex vivo methylene blue injection as a novel technique to improve lymph node visualization. [German]." Pathologe **29**(4): 274-279.
206. Marshall, E. K. (1942). "Chemotherapy of avian malaria." Physiol Rev **22**(2): 190-204.
207. Martinez Perez, J. L. and P. Hadad Melendez (1989). "Primaquine-Induced Hemolytic Syndrome and Glucose 6 Phosphate Dehydrogenase Deficiency." Revista Cubana de Medicina Tropical **41**(2): 299-306.
208. Mason, P. J., J. M. Bautista and F. Gilsanz (2007). "G6PD deficiency: the genotype-phenotype association." Blood Reviews **21**(5): 267-283.
209. McDonagh, E. M., J. M. Bautista, I. Youngster, R. B. Altman and T. E. Klein (2013). "PharmGKB summary: Methylene blue pathway." Pharmacogenetics and Genomics **23**(9): 498-508.
210. Mefane, C., D. R. Lenoble, D. Gendrel and E. Engonah (1985). "Diarrhea in the Young Child in Libreville Gabon Etiological Studies." Afrique Medicale **24**(234): 473-478.
211. Mehta, A., P. J. Mason and T. J. Vulliamy (2000). "Glucose-6-phosphate dehydrogenase deficiency." Best Practice and Research: Clinical Haematology **13**(1): 21-38.
212. Mehta, A. B. (1994). "Glucose-6-phosphate dehydrogenase deficiency." Prescribers' Journal **34**(5): 178-182.
213. Meierjohann, S., R. D. Walter and S. Muller (2002). "Regulation of intracellular glutathione levels in erythrocytes infected with chloroquine-sensitive and chloroquine-resistant Plasmodium falciparum." Biochem J **368**(Pt 3): 761-768.
214. Meissner, P. E., G. Mandi, S. Witte, B. Coulibaly, U. Mansmann, J. Rengelshausen, W. Schiek, A. Jahn, M. Sanon, T. Tapsoba, I. Walter-Sack, G. Mikus, J. Burhenne, K. D. Riedel, H. Schirmer, B. Kouyate and O. Muller (2005). "Safety of the methylene blue plus chloroquine combination in the treatment of uncomplicated falciparum malaria in young children of Burkina Faso [ISRCTN27290841]." Malaria Journal **4 (no pagination)**(45).

215.Melchior, E. (1946). "Surgery in Turkey. [German, English]." Ars Medici **36**(2): 675-680.
216.Mendoza, J. B. (1952). "Present concepts in malaria and its therapy." Journal of the Philippine Medical Association **28**(4): 190-213.
217.Miguel-Blanco, C., J. Lelievre, M. J. Delves, A. I. Bardera, J. L. Presa, M. J. Lopez-Barragan, A. Ruecker, S. Marques, R. E. Sinden and E. Herreros (2015). "Imaging-based high-throughput screening assay to identify new molecules with transmission-blocking potential against Plasmodium falciparum female gamete formation." Antimicrob Agents Chemother **59**(6): 3298-3305.
218.Mitchard, M. (1988). "Sulphur compounds used in medicine." Drug Metabolism and Drug Interactions **6**(3-4): 183-202.
219.Mizukawa, Y., J. F. Ge, A. Bakar Md, I. Itoh, C. Scheurer, S. Wittlin, R. Brun, H. Matsuoka and M. Ihara (2014). "Novel synthetic route for antimalarial benzo[a]phenoxazine derivative SSJ-183 and two active metabolites." Bioorganic and Medicinal Chemistry **22**(14): 3749-3752.
220.Mohanty, D., M. B. Mukherjee and R. B. Colah (2004). "Glucose-6-phosphate dehydrogenase deficiency in India." Indian Journal of Pediatrics **71**(6): 525-529.
221.Mohr, H., U. Pohl, B. Lambrecht, J. U. Wieding and H. Schmitt (1993). "Treatment of virus inactivated human plasma by means of methylene blue and light: Production and present clinical experiences. [German]." Infusionstherapie und Transfusionsmedizin **20**(SUPPL. 2): 19-24.
222.Mohring, F., J. Pretzel, E. Jortzik and K. Becker (2014). "The redox systems of Plasmodium falciparum and Plasmodium vivax: comparison, in silico analyses and inhibitor studies." Curr Med Chem **21**(15): 1728-1756.
223.Motau, H., R. Van Zyl and L. Harmse (2010). "Focused Conference Group: P01 - Clinical pharmacology in the emerging countries the chemotherapeutic action of synthetic dyes against Plasmodium falciparum." Basic and Clinical Pharmacology and Toxicology **107**: 472-473.
224.Mulay, H. D., T. D. Murthy, S. M. Nerune and M. R. Amrutha (2017). "New methylene blue stain for malaria detection on thin smears." Journal of Krishna Institute of Medical Sciences University **6**(1): 76-81.
225.Muller, D. (1931). "Glykoseoxydase. IV. Glykoseoxy-dase aus Aspergillus niger. Verhalten gegen Disaccharide (Maltoseoxydase), Glykuronsaure und Athylalkohol; Versuche mit Methylenblau und Monojodessigsaure." Biochem Zeitschr **232**(4/6): 423-434.
226.Muller, O., P. Meissner and U. Mansmann (2012). "Glucose-6-phosphate dehydrogenase deficiency and safety of methylene blue." Drug Safety **35**(1): 85.
227.Muller, O., F. P. Mockenhaupt, B. Marks, P. Meissner, B. Coulibaly, R. Kuhnert, H. Buchner, R. H. Schirmer, I. Walter-Sack, A. Sie and U. Mansmann (2013). "Haemolysis risk in methylene blue treatment of G6PD-sufficient and G6PD-deficient West-African children with uncomplicated falciparum malaria: a synopsis of four RCTs." Pharmacoepidemiol Drug Saf **22**(4): 376-385.
228.Muller, O., A. Sie, P. Meissner, R. H. Schirmer and B. Kouyate (2009). "Artemisinin resistance on the Thai-Cambodian border." The Lancet **374**(9699): 1419.
229.Muller, T., L. Johann, B. Jannack, M. Bruckner, D. A. Lanfranchi, H. Bauer, C. Sanchez, V. Yardley, C. Deregnaucourt, J. Schrevel, M. Lanzer, R. H. Schirmer and E. Davioud-Charvet (2011). "Glutathione Reductase-Catalyzed Cascade of Redox Reactions To Bioactivate Potent Antimalarial 1,4-Naphthoquinones - A New Strategy to Combat Malarial Parasites." Journal of the American Chemical Society **133**(30): 11557-11571.
230.Mutzke, E. (1962). "Comparison of reduction tests with resazurine and methylene blue purity tests and Koch's determination of germ contents for unprocessed milk." Deutsche Milchwirtschaft **9**(4): 100-101.
231.Mwangi, V. I., R. M. Mumo, D. M. Kiboi, S. A. Omar, Z. W. Ng'ang'a and H. S. Ozwara (2016). "Methylene blue inhibits lumefantrine-resistant Plasmodium berghei." J Infect Dev Ctries **10**(6): 635-642.
232.Nadiger, M., S. Shankar and S. Jain (2015). "Anti-malarial induced methemoglobinemia in a case of G6PD deficiency with malaria." Pediatric Blood and Cancer **62**: S66-S67.
233.Nagel, A. (1930). "Vital staining of fibrocytes of the adult rabbit grown in vitro." Zeitschr Wiss Biol Abt B Zeitschr Zellforsch U Mikrosk Anat **10**(4): 744-755.
234.Nepveu, F., E. Najahi and A. Valentin (2014). "Antimalarial activities of indolones and derivatives." Current Topics in Medicinal Chemistry **14**(14): 1643-1652.
235.Nepveu, F. and F. Turrini (2013). "Targeting the redox metabolism of Plasmodium falciparum." Future Medicinal Chemistry **5**(16): 1993-2006.
236.Nneji, C. M., O. A. Adaramoye, C. O. Falade and O. G. Ademowo (2013). "Effect of chloroquine, methylene blue and artemether on red cell and hepatic antioxidant defence system in mice infected with Plasmodium yoelii nigeriensis." Parasitol Res **112**(7): 2619-2625.
237.Nurr, J. (1943). "Methylene blue[long dash]an effective drug in dermatites, tonsilitis, and in fowl diphtheria." Berliner U Munchener Tierarztl Wochenschr **39**(40): 338-341.

238. O'Neill, P. M., V. E. Barton, S. A. Ward and J. Chadwick (2012). 4-aminoquinolines: Chloroquine, amodiaquine and next-generation analogues. Treatment and Prevention of Malaria : Antimalarial Drug Chemistry, Action and Use. H. M. Staines and S. Krishna. Switzerland, Springer Basel**:** 19-44.
239. O'Riordan, S., T. T. Hien, K. Miles, A. Allen, N. N. Quyen, N. Q. Hung, D. Q. Anh, L. N. Tuyen, D. B. Khoa, C. Q. Thai, D. M. Triet, N. H. Phu, S. Dunstan, T. Peto, J. Clegg, J. Farrar and D. Weatherall (2010). "Large scale screening for haemoglobin disorders in southern Vietnam: Implications for avoidance and management." British Journal of Haematology **150**(3): 359-364.
240. Obregon, D., E. C. Parker-Athill, J. Tan and T. Murphy (2012). "Psychotropic effects of antimicrobials and immune modulation by psychotropics: Implications for neuroimmune disorders." Neuropsychiatry **2**(4): 331-343.
241. Oguike, M. C. and G. O. Ademowo (2009). "Effect of chloroquine, methylene blue and artemether on hepatic oxidative stress and antioxidant enzymes in Plasmodium yoelii-infected mice." American Journal of Tropical Medicine and Hygiene **1)**: 39-40.
242. Ohrt, C., Q. Li, N. Obaldia, R. Im-Erbsin, L. Xie and J. Berman (2014). "Efficacy of intravenous methylene blue, intravenous artesunate, and their combination in preclinical models of malaria." Malaria journal **13**: 415.
243. Okombo, J., S. M. Kiara, L. Mwai, L. Pole, E. Ohuma, L. I. Ochola and A. Nzila (2012). "Baseline in vitro activities of the antimalarials pyronaridine and methylene blue against Plasmodium falciparum isolates from Kenya." Antimicrob Agents Chemother **56**(2): 1105-1107.
244. Olliaro, P. and T. N. C. Wells (2009). "The global portfolio of new antimalarial medicines under development." Clinical Pharmacology and Therapeutics **85**(6): 584-595.
245. On-Paew, P., T. Chadlane and P. Santanirand (2015). "Inhibition of Pythium insidiosum growth by methylene blue." Mycoses **58**: 67.
246. Orth, K., A. Ruck, G. Beck, A. Stanescu and H. G. Beger (1995). "Photodynamic therapy of small adenocarcinomas with methylene blue. [German]." Der Chirurg; Zeitschrift fur alle Gebiete der operativen Medizen **66**(12): 1254-1257.
247. Osterwald, A., J. Scholz, A. Gehl and K. Puschel (2012). "Differential diagnosis: Blue gastric mucosa: Post-mortem staining of the gastric mucosa with methylene blue. [German]." Archiv fur Kriminologie **230**(1-2): 35-41.
248. Oz, M., D. Isaev, D. E. Lorke, M. Hasan, G. Petroianu and T. S. Shippenberg (2012). "Methylene blue inhibits function of the 5-HT transporter." Br J Pharmacol **166**(1): 168-176.
249. Oz, M., D. E. Lorke, M. Hasan and G. A. Petroianu (2011). "Cellular and molecular actions of Methylene Blue in the nervous system." Medicinal Research Reviews **31**(1): 93-117.
250. Pal, C. and U. Bandyopadhyay (2012). "Redox-Active Antiparasitic Drugs." Antioxidants & Redox Signaling **17**(4): 555-582.
251. Pastore, S. (1929). "A special method for staining malarial parasites." Atti R Accad Naz Lincei Rend Cl Sci Fis Mat E Nat **9**(8): 669-671.
252. Pastrana-Mena, R., R. R. Dinglasan, B. Franke-Fayard, J. Vega-Rodriguez, M. Fuentes-Caraballo, A. Baerga-Ortiz, I. Coppens, M. Jacobs-Lorena, C. J. Janse and A. E. Serrano (2010). "Glutathione reductase-null malaria parasites have normal blood stage growth but arrest during development in the mosquito." J Biol Chem **285**(35): 27045-27056.
253. Penna-Coutinho, J., M. J. Almela, C. Miguel-Blanco, E. Herreros, P. M. Sa, N. Boechat and A. U. Krettli (2016). "Transmission-blocking potential of MEFAS, a hybrid compound derived from artesunate and mefloquine." Antimicrobial Agents and Chemotherapy **60**(5): 3145-3147.
254. Peter, S., E. Reichart, L. Poyntner and S. Mennel (2013). "Accidental staining of corneal nerves by methylene blue." Ophthalmologe **110**(9): 869-871.
255. Peters, A. L. and C. J. F. Van Noorden (2009). "Glucose-6-phosphate dehydrogenase deficiency and malaria: Cytochemical detection of heterozygous G6PD deficiency in women." Journal of Histochemistry and Cytochemistry **57**(11): 1003-1011.
256. Peters, W. (1999). "The evolution of tafenoquine - Antimalarial for a new millennium?" Journal of the Royal Society of Medicine **92**(7): 345-352.
257. Piane, L., M. L. Theron, M. Aumann and C. Trumel (2016). "Spurious reticulocyte profiles in a dog with babesiosis." Vet Clin Pathol **45**(4): 594-597.
258. Plouffe, D. M., M. Wree, A. Y. Du, S. Meister, F. W. Li, K. Patra, A. Lubar, S. L. Okitsu, E. L. Flannery, N. Kato, O. Tanaseichuk, E. Comer, B. Zhou, K. Kuhen, Y. Y. Zhou, D. Leroy, S. L. Schreiber, C. A. Scherer, J. Vinetz and E. A. Winzeler (2016). "High-Throughput Assay and Discovery of Small Molecules that Interrupt Malaria Transmission." Cell Host & Microbe **19**(1): 114-126.
259. Pradines, B., J. Dormoi, M. Madamet, B. Fall and R. Amalvict (2015). "Proveblue (methylene blue): A promising antimalarial drug." American Journal of Tropical Medicine and Hygiene **93 (4 Supplement)**: 467.
260. Quadbeck, G. and K. Randerath (1955). "Effect of concussion of the brain on the passage of methylene blue from the blood into the brain in the cat." ZEITSCHR NATURFORSCH. 10b.

261. Radi, A. E., H. M. Nassef and M. I. Attallah (2015). "Investigation of antimalarial drug pyrimethamine and its interaction with dsDNA by electrochemical and spectroscopic techniques." Analytical Methods **7**(10): 4159-4167.
262. Radosavljevi, A. and L. Ristl (1926). "Sedimentation rate and albumin content of the blood in malaria." Zeitschr Ges Exp Med **51**(1/2): 48-80.
263. Rahlfs, S. and K. Becker (2006). "Interference with redox-active enzymes as a basis for the design of antimalarial drugs." Mini-Reviews in Medicinal Chemistry **6**(2): 163-176.
264. Raman, J., S. Mody, R. P. Khubchandani, R. K. Mondal, U. Mann and M. Sharma (2003). "Primaquine induced methemoglobinemia [2] (multiple letters)." Indian Journal of Pediatrics **70**(6): 521-522.
265. Ramos, M. (1956). "Methylene blue in vesicular infestation due to Giardia. [Portuguese]." Revista Brasileira de Medicina **13**(2): 88-89.
266. Rauh, W. and K. Hammje (1983). "Determination of hydrogen sulphide in atmospheric air improvements of the procedure with methylene blue. [German]." Zeitschrift fur die Gesamte Hygiene und Ihre Grenzgebiete **29**(3): 159-162.
267. Rengelshausen, J., J. Burhenne, M. Fröhlich, Y. Tayrouz, S. Singh, K. Riedel, O. Müller, T. Hoppe-Tichy, W. Haefeli, G. Mikus and I. Walter-Sack (2004) "Pharmacokinetic interaction of chloroquine and methylene blue combination against malaria." European journal of clinical pharmacology **60**, 709-715 DOI: 10.1007/s00228-004-0818-0.
268. Rentz, E. (1940). "Methylenblau und Cholinesterase." Arch Exp Path U Pharmakol **196**: 148-160.
269. Rentz, E. (1941). "Choline-esterase in anaphyl-actic shock and the action of methylene blue." Arch Exptl Path U Pharmakol **198**: 385-389.
270. Rewerk, S., R. Braun, E. M. Stump and H. Thiele (1994). "In vivo staining of an enlarged parathyroid gland by methylene blue during reoperative neck surgery. [German]." Aktuelle Chirurgie **29**(1): 18-20.
271. Rigdon, R. H. and D. Breslin (1950). "Observations on the effect of certain of the antimalarial drugs on erythrocytes." Texas Repts Biol And Med **8**(3): 371-383.
272. Riggert, J., A. Humpe, G. Simson, K. Gutensohn and M. Kohler (1996). "Reduction of the methylene blue (MB) concentration in MB-photooxidated plasma by filtration. [German]." Infusionstherapie und Transfusionsmedizin **23**(6): 252-255.
273. Rodrigues, M. O., M. V. de Paula, K. A. Wanderley, I. B. Vasconcelos, S. Alves and T. A. Soares (2012). "Metal organic frameworks for drug delivery and environmental remediation: A molecular docking approach." International Journal of Quantum Chemistry **112**(20): 3346-3355.
274. Roe, M. A., A. Wilcox and R. D. Lillie (1906). "Eosinates of the azures and methylene blue in preparation of a satisfactory Giemsa stain from dyes of American manufacture." Publ Health Repts **56**(39): 1906-1909.
275. Rounds, D. E., W. Opel, R. S. Oison and I. V. Sheerman (1968). "The potential use of laser energy in the management of malaria." Biochemical and Biophysical Research Communications **32**(4): 616-623.
276. Russo, A., P. T. Donaldson and R. D. Lillie (1978). "Lower Azure B Methylene Blue Rations in Giemsa Type Blood and Malaria Stains." Stain Technology **53**(1): 37-42.
277. Sabalitschka, T. and A. Priem (1941). "Zur Bestimmung von Vitamin C. I. Unbrauch-barkeit der Vitamin-C-Bestimmung mit Methylenblau fiir Trockengemuse." Hoppe Seyler S Zeitschr Physiol Chem **270**(3/4): 194-200.
278. Sahlin, B. O. (1926). "The antagonism between methylene blue and KCN." Skand Arch Physiol **47**(6): 284-291.
279. Sanders, N. G., D. J. Sullivan, G. Mlambo, G. Dimopoulos and A. K. Tripathi (2014). "Gametocytocidal Screen Identifies Novel Chemical Classes with Plasmodium falciparum Transmission Blocking Activity." Plos One **9**(8): 13.
280. Sarbeen, J. I. and G. Sethu (2015). "Glucose-6-phosphate dehydrogenase deficiency." Research Journal of Pharmacy and Technology **8**(6): 792-795.
281. Sasoni, N., A. A. Iglesias, S. A. Guerrero and D. G. Arias (2016). "Functional thioredoxin reductase from pathogenic and free-living Leptospira spp." Free Radical Biology and Medicine **97**: 1-13.
282. Scheindlin, S. (2008). "Something old... something blue." Molecular Interventions **8**(6): 268-273.
283. Schenck, F. (1949). "The effect of atropine, 1-hyoscyamine, scopolamine, cocaine, choline, methylene blue, caffeine, histidine and dihy-droergotamine on the intraocular pressure of rabbits." Ophthalmologica **118**(1): 42-65.
284. Scheuch, D., K. H. Jacobasch, A. Hanel and C. Wagenknecht (1963). "Effect of methylene blue on glutathione and activity of some sh-enzymes. [German]." Acta Biol.Med.Germ **11**(5): 150-155.
285. Schirmer, R. H., H. Adler, M. Pickhardt and E. Mandelkow (2011). ""Lest we forget you - methylene blue..."." Neurobiology of Aging **32**(12): 2325.e2327-2325.e2316.
286. Schirmer, R. H., B. Coulibaly, A. Stich, M. Scheiwein, H. Merkle, J. Eubel, K. Becker, H. Becher, O. Mueller, T. Zich, W. Schiek and B. Kouyate (2003). "Methylene blue as an antimalarial agent." Redox Report **8**(5): 272-275.

287. Schmidt, D. and J. Haym (1968). "On the problem of a selective thermosensitization of carcinoma cells in vivo with vitamin K3-sodium bisulfite, methylene blue and other thermosensitizators. 1. On the toxicology of vitamin K3-sodium bisulfite in combination with methylene blue on rats and dogs. [German]." Arzneimittel-Forschung **18**(6): 676-682.
288. Schneider, H. J., A. Hesse, W. Berg, U. Lange, U. Hartmann and K. Hensel (1977). "Nutritional influences on composition of foreign body stones in animal experiments. III. Cation exchangers, anthroquinone derivatives, diphosphonates and methylene blue. [German]." Zeitschrift fur Urologie und Nephrologie **70**(5): 357-362.
289. Schneider, P. and R. Link (1962). "Comparative analysis with the methylene blue, resazurine and nitrite reduction test in unprocessed milk." Deutsche Milchwirtschaft **9**(2): 34-36.
290. Schott, E. (1926). "Experimental influence of the form of the electrocardiogram. The change of the form of the electrocardiogram under the action of acid, alkali, and methy-lene blue." Deutsches Archiv Fur Klin Med **152**(5/6): 287-301.
291. Schrader, F. C., M. Barho, I. Steiner, R. Ortmann and M. Schlitzer (2012). "The antimalarial pipeline - An update." International Journal of Medical Microbiology **302**(4-5): 165-171.
292. Schrauzer, G. N. and W. J. Michaely (1972). "Methylene blue as an inhibitor of the reduction of hydroxocobalamin by carbon monoxide. [German]." Zeitschrift fur Naturforschung **Teil B. Anorganische Chemie, organische Chemie, Biochemie, Biophysik, Biologie. 27**(5): 577-578.
293. Schubert, R. H. W. and J. G. Esanu (1977). "The methyleneblue reduction disc test (M.R.D. Test): a simplified method for the identification of Pseudomonas species. [German]." Zentralblatt fur Bakteriologie Mikrobiologie und Hygiene - Abt **1 Orig. A. 239**(4): 504-509.
294. Schubert, R. H. W., J. G. Esanu and F. Esanu (1975). "The methylene blue reduction test (MR test) and the micro TTC test for the determination of substances as sole source of carbon in the taxonomy of Pseudomonas species. [German]." Zbl.Bakt.Reihe B **160**(1): 50-59.
295. Schultze, M., S. Rapoport and A. Lach (1965). "Effect of methylene blue on the metabolism of substrate in the reticulocyte. [German]." Folia haematologica (Leipzig, Germany : 1928) **83**(4): 477-484.
296. Scotti, L., F. J. Filho, R. O. De Moura, F. F. Ribeiro, H. Ishiki, M. S. Da Silva, J. Filho and M. T. Scotti (2016). "Multi-Target Drugs for Neglected Diseases." Current Pharmaceutical Design **22**(21): 3135-3163.
297. Senarathna, G., M. Page-Sharp and A. Crowe (2014). "In vitro drug transport of antimalarials; amodiaquine, mefloquine and methylene blue SMDK." American Journal of Tropical Medicine and Hygiene **1)**: 71.
298. Senarathna, S., M. Page-Sharp and A. Crowe (2016). "The Interactions of P-Glycoprotein with Antimalarial Drugs, Including Substrate Affinity, Inhibition and Regulation." Plos One **11**(4): 17.
299. Shakespeare, P. G., P. I. Trigg, S. I. Kyd and L. Tappenden (1979). "Glucose Metabolism in the Simian Malaria Parasite Plasmodium-Knowlesi Activities of the Glycolytic and Pentose Phosphate Pathways during the Intra Erythrocytic Cycle." Annals of Tropical Medicine & Parasitology **73**(5): 407-416.
300. Shanks, D. (2014). "Drugs for malaria elimination: What do we have now and what do we need?" Malaria Journal **13**: S8-S9.
301. Sharma, N. and S. Varma (2003). "Unusual life-threatening adverse drug effects with chloroquine in a young girl [2]." Journal of Postgraduate Medicine **49**(2): 187.
302. Sherndal, A. E. (1943). "Chemistry and development of atabrine and plasmochin. Chem. and Engineer." News **21**(14): 1154-1158.
303. Siciliano, G., T. R. Santha Kumar, R. Bona, G. Camarda, M. M. Calabretta, L. Cevenini, E. Davioud-Charvet, K. Becker, A. Cara, D. A. Fidock and P. Alano (2017). "A high susceptibility to redox imbalance of the transmissible stages of Plasmodium falciparum revealed with a luciferase-based mature gametocyte assay." Mol Microbiol.
304. Sidorov, P., I. Desta, M. Chesse, D. Horvath, G. Marcou, A. Varnek, E. Davioud-Charvet and M. Elhabiri (2016). "Redox Polypharmacology as an Emerging Strategy to Combat Malarial Parasites." Chemmedchem **11**(12): 1339-1351.
305. Sikka, P., V. K. Bindra, S. Kapoor, V. Jain and K. K. Saxena (2011). "Blue cures blue but be cautious." Journal of Pharmacy and Bioallied Sciences **3**(4): 543-545.
306. Singh, S. K. and S. Singh (2014). "A brief history of quinoline as antimalarial agents." International Journal of Pharmaceutical Sciences Review and Research **25**(1): 295-302.
307. Sneader, W. (2002). "The 50th anniversary of chlorpromazine." Drug News and Perspectives **15**(7): 466-471.
308. Sorgel, F. (2004). "The return of Ehrlich's 'Therapia magna sterilisans' and other Ehrlich concepts?. Series of papers honoring Paul Ehrlich on the occasion of his 150th birthday." Chemotherapy **50**(1): 6-10.

309. Stanford, S. C., B. J. Stanford and P. K. Gillman (2010). "Risk of severe serotonin toxicity following co-administration of methylene blue and serotonin reuptake inhibitors: an update on a case report of post-operative delirium." J Psychopharmacol **24**(10): 1433-1438.
310. Stauffacher, H. (1927). "The causative agent of foot-and-mouth disease." Rev Suisse Zool **34**(2): 207-216.
311. Steiner, H. H. and H. G. Steiner-Milz (1986). "Severe complications after intrathecal application of methylene blue. [German]." Laryngologie Rhinologie Otologie **65**(12): 699-701.
312. Stormer, U. and H. Baumgartel (1986). "Water-induced metachromasia of thionine and methylene blue in histologic preparations. [German]." Acta histochemica **79**(1): 127-134.
313. Sullivan, D. J. (2013). "Plasmodium drug targets outside the genetic control of the parasite." Curr Pharm Des **19**(2): 282-289.
314. Suwanarusk, R., B. Russell, A. Ong, K. Sriprawat, C. S. Chu, A. PyaePhyo, B. Malleret, F. Nosten and L. Renia (2015). "Methylene blue inhibits the asexual development of vivax malaria parasites from a region of increasing chloroquine resistance." J Antimicrob Chemother **70**(1): 124-129.
315. Tajeddin, L. and H. Baseri (2009). "Study on fungal flora in midgut of larvae and adult of malaria vector, Anopheles stephensi." Tropical Medicine and International Health **14**: 159.
316. Teguh, S. C., N. Klonis, S. Duffy, L. Lucantoni, V. M. Avery, C. A. Hutton, J. B. Baell and L. Tilley (2013). "Novel conjugated quinoline-indoles compromise Plasmodium falciparum mitochondrial function and show promising antimalarial activity." Journal of Medicinal Chemistry **56**(15): 6200-6215.
317. Toepfer, K. (1970). "Spectrophotometric and histochemical qualities of 1:9 dimethyl methylene blue as a metachromatic dye. [German]." Histochemie **Histochemistry. Histochimie. 21**(1): 64-72.
318. Tschochner, H. J., H. Gohler and G. Fruhauf (1980). "Experiences with the methyl blue reduction Tes Mikruvid I for the recognition of bacteriurias. [German]." Zeitschrift fur Urologie und Nephrologie **73**(9): 681-686.
319. Tyagi, C., J. Bathke, S. Goyal, M. Fischer, H. M. Dahse, S. Chacko, K. Becker and A. Grover (2015). "Targeting the intersubunit cavity of Plasmodium falciparum glutathione reductase by a novel natural inhibitor: Computational and experimental evidence." International Journal of Biochemistry & Cell Biology **61**: 72-80.
320. Tyski, S. (2003). "Non-antibiotics - Drugs with additional antimicrobial activity." Acta Poloniae Pharmaceutica - Drug Research **60**(5): 401-404.
321. Uh, L. D. (2011). "Serotonergic psychotropic agents: Serotonin syndrome with linezolid and methylene blue. [German]." Deutsche Apotheker Zeitung **151**(31): 34-35.
322. Urban, P. and X. Fernandez-Busquets (2014). "Nanomedicine against malaria." Current Medicinal Chemistry **21**(5): 605-629.
323. Van Den Berg, F. W. J. (1977). "Erythrocyte glucose 6 phosphate dehydrogenase deficiency. [Dutch]." Tijdschrift voor Geneeskunde **33**(24): 1391-1401.
324. van Pelt-Koops, J. C., H. E. Pett, W. Graumans, M. van der Vegte-Bolmer, G. J. van Gemert, M. Rottmann, B. K. S. Yeung, T. T. Diagana and R. W. Sauerwein (2012). "The Spiroindolone Drug Candidate NITD609 Potently Inhibits Gametocytogenesis and Blocks Plasmodium falciparum Transmission to Anopheles Mosquito Vector." Antimicrobial Agents and Chemotherapy **56**(7): 3544-3548.
325. Vennerstrom, J. L., M. T. Makler, C. K. Angerhofer and J. A. Williams (1995). "Antimalarial dyes revisited: Xanthenes, azines, oxazines and thiazines." Antimicrobial Agents and Chemotherapy **39**(12): 2671-2677.
326. von Ardenne, M. and P. G. Reitnauer (1967). "The superadditive potentation of the vitamin K-induced selective thermosensitization of cancer cells by methylene blue. [German]." Zeitschrift fur Krebsforschung **70**(2): 165-171.
327. von Ardenne, M., P. G. Reitnauer and D. Schmidt (1967). "On the toxicology of vitamin K3-sodium bisulfite and its combination with methylene blue. [German]." Arzneimittel-Forschung **17**(11): 1339-1346.
328. Wachtel, D. and E. Russwurm (1972). "A new possibility in the diagnosis of bacteriurias using methylene blue reduction in vivo. [German]." Das Deutsche Gesundheitswesen **27**(20): 949-952.
329. Wagner-Romero, F., J. Convit, E. Bernt and M. Nelbock-Hochstetter (1966). "The photooxydation of nlcotinamide-adenlne-dinucleotide and of nlcotinamide-adenine-dinucleotidephosphate using methylene blue [Engl. summ.]." Biochem Z **346**(2): 167-170.
330. Wainwright, M. (2005). "The development of phenothiazinium photosensitisers." Photodiagnosis Photodyn Ther **2**(4): 263-272.
331. Wainwright, M. and M. S. Baptista (2011). "The application of photosensitisers to tropical pathogens in the blood supply." Photodiagnosis and Photodynamic Therapy **8**(3): 240-248.
332. Walter-Sack, I., J. Rengelshausen, H. Oberwittler, J. Burhenne, O. Mueller, P. Meissner and G. Mikus (2009) "High absolute bioavailability of methylene blue given as an aqueous oral formulation." European journal of clinical pharmacology **65**, 179-189 DOI: 10.1007/s00228-008-0563-x.

333. Wang, J. Q., C. Y. Zhao, P. Kong, G. Y. Bian, Z. Sun, Y. F. Sun, L. Guo and B. Li (2016). "Methylene blue alleviates experimental autoimmune encephalomyelitis by modulating AMPK/SIRT1 signaling pathway and Th17/Treg immune response." Journal of Neuroimmunology **299**: 45-52.
334. Warburg, O., F. Kubowitz and W. Christian (1930). "Uber die katalytische Wirkung von Methylenblau in lebenden Zellen." Biochem Zeitschr **227**(4/6): 245-271.
335. Watanabe, A. (1932). "Uber die Beeinflussung der Atmung von einigen grunen Algen durch Kaliumcyanid und Methylenblau. Beitrage zur Stoffwechselphysiologie der Algen. I." Acta Phytochim **6**(2): 315-335.
336. Waters, N. C. and M. D. Edstein (2012). 8-aminoquinolines: Primaquine and tafenoquine. Treatment and Prevention of Malaria : Antimalarial Drug Chemistry, Action and Use. H. M. Staines and S. Krishna. Switzerland, Springer Basel**:** 69-94.
337. Weiland, E. (1944). "Untersuchungen uber die redu-zierende Wirkung von Ascorbinsaure auf Methylenblau bei Belichtung." Biochem Zeitschr **317**(3/4): 168-170.
338. Wells, T. N. C. (2012). New medicines to combat malaria: An overview of the global pipeline of therapeutics. Treatment and Prevention of Malaria : Antimalarial Drug Chemistry, Action and Use. H. M. Staines and S. Krishna. Switzerland, Springer Basel**:** 227-247.
339. Wendel, W. B. (1943). "Respiratory and carbohydrate metabolism of malaria parasites (Plasmodium knowlesi)." Jour Biol Chem **148**(1): 21-34.
340. Wendt, E. (1964). "The influence of thyroxine and methylene blue on the indirect radiation effect in chick embryos. [German]." Atompraxis **10**(2): 114-118.
341. Westphal, A. (1951). "The dependence of the titre grade of Sabin-Feldman's test on the methylene-blue used. [German]." Zeitschrift fur Tropenmedizin und Parasitologie **3**(1): 72-77.
342. Wieding, J. U. and H. Neumeyer (1992). "First experience with methylene blue virus-inactivated fresh frozen plasma: Results of a clinical and an in vitro study. [German]." Infusionstherapie und Transfusionsmedizin **19**(2): 84-88+90.
343. Wieland, H. and O. B. Claren (1934). "Die Dehydrierung durch Hefe bei Gegenwart von Methylenblau und Chinon. Uber den Mechanismus der Oxydationsvorgange. XXXIX." Justus Liebigs Ann Chem **509**(2): 182-200.
344. Williams, M. (2000). "Special issue - Non antibiotics." International Journal of Antimicrobial Agents **14**(3): 171.
345. Wirjanata, G., B. F. Sebayang, F. Chalfein, Prayoga, I. Handayuni, L. Trianty, E. Kenangalem, R. Noviyanti, B. Campo, J. R. Poespoprodjo, J. J. Mohrle, R. N. Price and J. Marfurt (2015). "Potent Ex Vivo Activity of Naphthoquine and Methylene Blue against Drug-Resistant Clinical Isolates of Plasmodium falciparum and Plasmodium vivax." Antimicrob Agents Chemother **59**(10): 6117-6124.
346. Wolff, J. W. (1948). "A simple and quick staining method for blood examination of malaria parasites. [Dutch]." Nederlands Tijdschrift voor Geneeskunde **3**(37): 2834-2837.
347. Wrenger, C., M. L. Eschbach, I. B. Muller, D. Warnecke and R. D. Walter (2005). "Analysis of the vitamin B6 biosynthesis pathway in the human malaria parasite Plasmodium falciparum." J Biol Chem **280**(7): 5242-5248.
348. Wu, R. W. K., H. W. Chan, M. K. Cheung, K. W. Lee, G. W. C. Chan and R. K. Haynes (2010). "From methylene blue to chloroquine to the modern artemisinin antimalarial artemisone: Development and clinical aspects." Medicinal Chemistry Research **19**: S19-S20.
349. Wydra, D., S. Sawicki, J. Emerich and G. Romanowicz (2005). "Evaluation of sentinel node detection in vulvar cancer." Nuclear Medicine Review **8**(2): 128-130.
350. Xie, L. K., W. J. Li, A. Winters, F. Yuan, K. L. Jin and S. H. Yang (2013). "Methylene blue induces macroautophagy through 5 ' adenosine monophosphate-activated protein kinase pathway to protect neurons from serum deprivation." Frontiers in Cellular Neuroscience **7**: 9.
351. Yeh, I. and R. B. Altman (2006). "Drug targets for Plasmodium falciparum: A post-genomic review/survey." Mini-Reviews in Medicinal Chemistry **6**(2): 177-202.
352. Youngster, I. and M. Berkovitch (2012). "Glucose-6-Phosphate Dehydrogenase Deficiency and Safety of Methylene Blue Reply." Drug Safety **35**(1): 85-86.
353. Zachee, P., H. Demuynck, G. Verhoef and M. A. Boogaerts (1994). "Hematological abnormalities in migrants. [Dutch]." Tijdschrift voor Geneeskunde **50**(10): 821-827.
354. Ziebuhr, W., K. Xiao, B. Coulibaly, R. Schwarz and T. Dandekar (2004). "Pharmacogenomic strategies against resistance development in microbial infections." Pharmacogenomics **5**(4): 361-379.
355. Ziegler, J., R. Linck and D. W. Wright (2001). "Heme aggregation inhibitors: Antimalarial drugs targeting an essential biomineralization process." Current Medicinal Chemistry **8**(2): 171-189.
356. Zirkel, J., A. Cecil, W. Heinz, B. Coulibaly, H. Schirmer and T. Dandekar (2011). "Thiol-dependent redox networks: Analysing the effects of the anti-malarial agent Methylene Blue." International Journal of Medical Microbiology **301**: 11-12.
357. Zirkel, J., A. Cecil, F. Schafer, S. Rahlfs, A. Ouedraogo, K. Xiao, S. Sawadogo, B. Coulibaly, K. Becker and T. Dandekar (2012). "Analyzing thiol-dependent redox networks in the presence of

methylene blue and other antimalarial agents with RT-PCR-supported in silico modeling." Bioinformatics and Biology Insights **6**: 287-302.
358. Zissopoulos, P., G. Vlassis and G. Karditsas (1978). "Rapid staining method with methylene blue for cytologic smears. [German]." Zentralblatt fur Gynakologie **100**(17): 1119-1122.
359. Zoungrana, A., B. Coulibaly, A. Sie, I. Walter-Sack, F. P. Mockenhaupt, B. Kouyate, R. H. Schirmer, C. Klose, U. Mansmann, P. Meissner and O. Muller (2008). "Safety and efficacy of methylene blue combined with artesunate or amodiaquine for uncomplicated falciparum malaria: A randomized controlled trial from Burkina Faso." PLoS ONE **3 (2)** (e1630).

## Part II Additional records identified through hand searching of all resources (n=115)

360. (2008). Reports from University of Heidelberg Department of Internal Medicine describe recent advances in malaria in children, University of Heidelberg.
361. (2009). "Blue is the colour: Letters to the editor." 21. 2015, from http://www.thetimes.co.uk/tto/opinion/letters/article2070075.ece.
362. Alving, A. S., et al. (1949). the clinical testing of antimalarial drugs at Stateville Pententiary: semi annual report 1948-1949. NIH Malaria Report, Antimalarial Grant No 198. **87**.
363. Anschütz (1910). "Einwirkung von Chinin und Methylenblau auf Protozoen. ." Zentralblatt für Bakteriologie **54**(3).
364. Apostolescu, c. M. a. (1926). "Malarial Parasites and Methylene Blue." The Lancet **208**(5372): 361.
365. Appel, L. (1917). "Zur Behandlung der Malaria mit Methylenblau und Salvarsan." Deutsche med. Wochenschr. **43**: 1359-1360.
366. Argutinsky (1902). "Malariastudien." Arch f mikr Anatomie **LIX, LXI**.
367. Atkinson (1903). Lancet **1**: 1370.
368. Baird, J. (1894). "Methylene blue mediated hexose monophosphate shunt stimulation in human red blood cells in vitro: independance from intracellular oxidative injury." Int. J. Biochem. **16**: 1053-1058.
369. Barber, M. A. (1832). "Malaria studies on the Firestone rubber plantation in Liberia, West Africa." Am J Hyg **15**: 601-633.
370. Beck (1890-1899). "Methylenblau en Chinine by Malaria." Genekundig Tijdschrift voor Nederlandsch-Indie **37**(5,6).
371. Beck, K. (1893). "Über die Behandlung der Malaria mit Methylenblau und dessen lokale Anwendung bei Diphtheritis." Centralblatt für Klinische Medizin **14**(25): 521-524.
372. Boignet and Trintignan (1892). "Du bleu de methylene dans le paludisme et dans la blenhorragie." Bull Med Paris **46**: 943-944.
373. Boshowsky (1909). Wratschebnaja Gazeta **41**.
374. Cardamatis (1901). "Forme très grave de cachexie paludéenne aigue." Progrés Medical.
375. Cardamatis, J. P. (1898). "275 klinische Beobachtungen über das Methylenblau." Deutsche medizinische Wochenschrift Therapeutische Beilage **2**: 9.
376. Celli (1900). Zentralblatt für Bakteriologie und Parasitenkunde **27**: 110.
377. Commission, t. G. M. (1899). "Berlin (from our own correspondent)." The Lancet **153**(3937): 411-412.
378. Dobrowski (1894, 28.01.). Gazetta Lekarska.
379. d'Oelsnitz (1911). Revue internationale de medecine et de chirurgie.
380. Dornblüth (1903). Die Arzneimittel der heutigen Medizin.
381. Drzwiechi, D. (1893). New-York Medical Record: 202.
382. Ehrlich, P. (1891). "Über die wirkung des methylenblau bei malaria."
383. Ferreira, M. C. (1893). "Sur l'emploi du bleu de méthylène dans la malaria infantile." Revue de Therapeutique Medico-Chirugicale **124**: 488-525.
384. Ferreira, M. C. (1893). "treatment of malaria with methylene blue." The Lancet **142**(3657): 821.
385. Flebbe (1919). "Über die Malaria im Taurus (Kleinasien)." Deutsche Medizinische Wochenschrift **45 II**(41): 1138-1140.
386. Floeckinger "Methylenblau in der gynäkologie."
387. Fratnich (1894). "Methylene Blue in Malaria." Therapeutische Monatshefte **4**: 150.
388. Frohlich, A. (1948). "Not Available. [Polyglot]." Wiener klinische Wochenschrift **60**(24): 379-381.
389. Glogner, P. (1899). "Ueber die im Malaischen Archipel vorkommende Malaria-Erreger nebst einigen Fieberkurven." 444-455.
390. Glogner, P. (1901). "Ein Beitrag zur Beurtheilung der Malaria Rezidive und ihrer Behandlung." 171-191.
391. Grabenko, I. K. (1946). "Combined methylene blue and glucose intravenous therapy of malaria " JAMA **132**(8): 481.
392. Grawitz (1892). Berliner Klinische Wochenschrift **2**: 138.

393. Greenwood, D. (1995). "Historical perspectives Conflict of interest: The genesis of synthetic anitmalarial agents in peace and war." J. Antimicrobial Chemotherapy **36**: 857-872.
394. Guttmann, P. (1893). "Über die Behandlung der Malaria mit Methylenblau." Deutsche Medizinische Wochenschrift **1**: 28.
395. Herbert (1903). Revue internationale de medecine et de chirurgie: 117.
396. Hunt, B. M. (1894). "The action of Methylene blue on Löffler's Bacilli, with clinical suggestions." The Lancet: 792-794.
397. Iwanoff, A. (1901). Über die Behandlung der Malaria mit Methylenblau. Moskau.
398. Kahane (1910). "Die Arzneitherapie der Gegenwart."
399. Kalberlah and Schloßberger (1918). "Chemo-therapeutische Studien bei chronischer Malaria." Deutsche med. Wochenschr. **40**.
400. Kasem-Beck (1893). "Über die Behandlung der Malaria mit Methylenblau." Zentralblatt für klinische Medizin **25**: 521.
401. Kaufmann, P. (1919). "On the effect of methylene blue in malaria." Deutsche med. Wochenschr. **45**: 1365.
402. Keponer.
403. Ketli, K. "Über die antimalarische Wirkung des Methylenblau " Ungarisches Archiv für Medizin **2**(1): 1-11.
404. Kleine (1902). "Über Schwarzwasserfieber." Zeitschrift f Hygiene **38**.
405. Koch (1902). Zeitschrift f Hygiene **30**.
406. Kothny (1914). Klinisch-therapeutische Wochenschrift: 580.
407. Laveran (1888). "Traité du paludisme."
408. Laveran (1907). Paludisme.
409. Lendle, L. (1961). "Die Therapeutische Anwendung Von Methylenblau." Deutsche Medizinische Wochenschrift **86**(10): 452-&.
410. Loewenberg, R. D. (1925). "Über die Wirkung von Methylenblau auf ein einheimisches Quartanfieber." Deutsche Medizinische Wochenschrift **51**(29): 1194-1195.
411. Marc (1903). "Die Malaria im Turkestan." 365-403.
412. Marchoux, E. (1897). "Le paludisme au Sénegal " Annaales de l'Institute Pasteur.
413. Marschall, D. G. (1920). "Notes short comments." The Lancet: 1334.
414. Marschall, D. G. and F. W. Gee (1894). "On the Use of Methylene Blue in Malarial Fevers." Indian Medical Gazette **28**: 409-410 bzw 452-453.
415. mayer, M. (1919). "The effect of methylene blue in cases of Malaria quartana." " Deutsche med. Wochenschr. **45**: 1052-1053.
416. Mayer, M. (1922). "Über die Behandlung der Malaria." " Klinische Wochenschrift **1**(11): 527-529.
417. Mays (1898). "Über die therapeutische Verwendbarkeit des Methylenblau." Münchener med Wochenschrift **24**: 745.
418. Michailow (1899). Petersburger medizinische Wochenschrift: 209.
419. Misclescu, A. and W. L. (2010). "Methylene blue, an old drug with new indications?" Jurnalul Romana de Anestezie Terapie Intensiva(17): 35-41.
420. Moncorvo (1895). Gazette hebdomadaire de Paris **47**.
421. Moore, J. T. (1902). "Methylene Blue and Quinine in Malaria." Medical News **LXXXI**: 1063.
422. Mühlens (1903). Deutsche medizinische Wochenschrift (1946): 627.
423. Mühlens (1910). "Über einheimische Malaria quartana." Deutsche Medizinische Wochenschrift **36 II**(42): 1948-1951.
424. Mühlens (1921). "Parasitologische und klinische Beobachtungen bei künstlichen Malaria und Recurrensübertragungen."
425. Mya, G. (1892). "Methylene blue in malaria." British medical journal: 1155.
426. Neumann (1893). Therapeutische Monatshefte: 190.
427. Neumann (1893). "Ueber die Wirkung des Methylenblau bei Malaria." Pester Med.-Chir. Presse **1**.
428. Nocht-Werner (1910). "Beobachtungen über relative Chininresistenz bei Malaria aus Brasilien." Deutsche med. Wochenschr.: 1557.
429. Noguera (1905). Revista de medicina y cirujia.
430. O.A.C., A. and A. C. Pinto (1984). "Malaria and antimalarial agents." Revista Brasileira de Farmacia **65**(1-3): 2-21.
431. Ollwig (1899). "Ein Beitrag zur Behandlung der Malaria mit Methylenblau." Zeitschrift f Hygiene **31**: 317-336.
432. Ortner Vorlesungen über spezifische Therapie innerer Krankheiten.
433. Otto (1902). "Ein in unseren Breiten erworbener Fall von Schwarzwasserfieber usw." Deutsche Medizinische Wochenschrift **4**.
434. Panse (1903). "Schwarzwasserfieber." 1-44.

435. Parenski and Blatteis (1893). "Über das Methylenblau bei Malaria." Therapeutische Monatshefte: 16-17.
436. Phillipi (1913). "Würzburger Abhandlungen aus den Gesamtgebieten der praktischen Medizin.".
437. Plehn (1892). "Beitrag zur Pathologie der Tropen." 285-309.
438. Plehn (1895). "Über das Schwarzwasserfieber an der afrikanischen Westküste." Deutsche Medizinische Wochenschrift **25, 26, 27**.
439. Plehn (1898). "Über die Kamerun-Küste."
440. Plehn (1901). "Über die praktischen Ergebnisse der neueren Malariaforschung usw." Deutsche Medizinische Wochenschrift: 46-49.
441. Reitler, R. (1920). Wiener klinische Wochenschrift **33**: 9.
442. Rengelshausen, J. e. a. (2004). "Pharmacokinetic interaction of chloroquine and methylene blue combination against malaria." European Journal of Clinical Pharmacology **60**: 709-715.
443. Reooter.
444. Rijken, M., et al. (2011). "Chloroquine resistant vivax malaria in a pregnant woman on the western border of Thailand." Malar J **10**: 113.
445. Riskin (1900). Eshenedelnik **48, 49**.
446. Romanowsky (1891). Zur Frage über die Parasitologie und Therapie der Malaria (russisch). St. Petersburg.
447. Rosin (1893). "Einfluss von Chinin und Methylenblau auf lebende Malariaparasiten." Deutsche Medizinische Wochenschrift(44): 1068-1070.
448. Roß-Thomson (1912). Annals of Tropical Medicine and Parasitology **5**.
449. Röttger, W. (1895). Ein Beitrag zur Behandlung der Malaria mit Methylenblau. Kiel, University Kiel**:** 28.
450. Röttger, W. (1896). "Zur Behandlung Malariakranker mit Methylenblau." Deutsche Medizinische Wochenschrift **15**: 237.
451. Rüge, R. (1906). Einführung in das Studium der Malariakrankheiten. Jena, G. Fischer.
452. Ruszinak, S. (1920). Wiener klinische Wochenschrift **33**.
453. Schlitzer, M. (2007). "Malaria chemotherapeutics part I: history of antimalarial drug development, durrently used therapeutics and drugs in clinical development." Chem. Med Chem **2**: 944-986.
454. Simons, R. (1919). "Malaria-Erfahrungen und kritische Studien über den Unitarismus." Berliner Klinische Wochenschrift **2**: 1009-1012.
455. Simons, R. (1920). Arch. f. Schiffs-+ Tropenhygiene: 206.
456. Sivers (1901). Finska Läkaresällskapets Handlingar **43**.
457. Smithwick (1900). Merck's Archives: 50.
458. Society, R. (1901). "Reports to the Malaria Commitee."
459. Steudel (1894). Die perniziöse Malaria in Deutsch-Ostafrika, F. C. W. Vogel,.
460. Stoppe.
461. Streicher (1908). Semaine medicale: 454.
462. Thayer, W. S. (1892). "On the Value of methylene blue in malarial fever." Bulletin of Johns Hopkins Hospital **3**(22): 49-54.
463. Thur (1893). Geneeskundige Tijdschrift voor· Nederlandsch Indie **33**.
464. Tsuzuki (1902). "Über die Ergebnisse meiner Malariaforschung in Hokkaido (Japan) " Centralblatt für Bakteriologie **15**.
465. Vagedes (1903). "Bericht über die Malariaexpedition in Deutsch-Südwestafrika." 83-132.
466. Valvasori-Peroni (1893). Gazzetta degli ospedali e delle cliniche **Nr.11ft.**
467. Voitel (1926). "über die Wirkung des Eosins bei der Abstellung der therapeutischen Impfmalaria." Klinische Wochenschrift **5**(45): 2117.
468. Wainwright, M. and K. B. Crossley (2002). "Methylene Blue - a therapeutic dye for all seasons?" Journal of Chemotherapy **14**(5): 431-443.
469. Wenzel (1905). Handbuch der Tropenkrankheiten. Leipzig, Ziemann, H.
470. Werner, E. R. (1911). Therapeutische Monatshefte.
471. WHO (2003). assessment and monitoring of antimalarial drug efficacy for the treatment of uncomplicated falciparum malaria, WHO/HTM/RBM. **50**.
472. Wood, C. (1904). "the use of methylene blue in malarial fevers." Proceedings of the Philadelphia County Medical Society.
473. Ziemann "Über Malaria und andere Blutparasiten ".
474. MERCK, E. 1922. Merck's Wissenschaftliche Abhandlungen aus den Gebieten der Pharmakotherapie, Pharmazie und verwandter Disziplinen-Anilinfarben in der Therapie, Darmstadt, Darmstadt.
